# Classification of Non-Infectious and/or Immune Mediated Choroiditis: A Brief Overview of the Essentials

**DOI:** 10.3390/diagnostics11060939

**Published:** 2021-05-24

**Authors:** Carl P. Herbort, Alessandro Mantovani, Ilknur Tugal-Tutkun, Ioannis Papasavvas

**Affiliations:** 1 Retinal and Inflammatory Eye Diseases, Centre for Ophthalmic Specialized Care (COS), Clinic Montchoisi Teaching Centre, 1003 Lausanne, Switzerland; i.s.papasavvas@gmail.com; 2 Department of Ophthalmology, Valduce Hospital, 22100 Como, Italy; aless.mant@gmail.com; 3 Department of Ophthalmology, Istanbul Faculty of Medicine, Istanbul University, 34093 Istanbul, Turkey; itutkun@yahoo.com

**Keywords:** non-infectious choroiditis, fluoresceine angiography, indocyanine angiography, MEWDS, APMPPE, multifocal choroiditis, Serpiginous choroiditis, Birdshot retinochoroiditis, VKH, sympathetic ophthalmia, sarcoidosis chorioretinitis

## Abstract

The choroid was poorly accessible to imaging investigation until the last decade of the last century. With the availability of more precise imaging methods such as indocyanine green angiography (ICGA) and, later, optical coherence tomography (OCT), enhanced depth OCT (EDI-OCT), and OCT angiography (OCTA), appraisal of choroidal inflammation has substantially gained in accuracy. This allowed to precisely determine which structures were touched in the different non-infectious choroiditis entities and made it possible to classify this group of diseases, ICGA signs, mainly hypofluorescent lesions, were identified and described. Previous publications have divided angiographic findings into two main sets of signs: (1) irregular “geographic” hypofluorescent areas corresponding to choriocapillaris non-perfusion and (2) round more regular, hypofluorescent dark dots more evenly distributed in the fundus corresponding to more deep choroidal stromal foci. These distinct findings allowed to subdivide and classify choroiditis into choriocapillaritis and stromal choroiditis. Additional signs were identified from EDI-OCT and OCTA examination supporting the classification of choroiditis into choriocapillaritis and stromal choroiditis. Results: Diseases involving principally the choriocapillaris included Multiple Evanescent White Dot Syndrome (MEWDS), Acute Posterior Multifocal Placoid Pigment Epitheliopathy (APMPPE), Idiopathic Multifocal Choroiditis (MFC), and Serpiginous Choroiditis (SC) as well as mixed forms. Diseases primarily involving the choroidal stroma included HLA-A29 Birdshot Retinochoroiditis (BRC), Vogt-Koyanagi-Harada disease (VKH), Sympathetic Ophthalmia (SO), and Sarcoidosis chorioretinitis (SARC). Thanks to new imaging investigations of the choroid, it is now possible to classify and understand the diverse clinicopathological mechanisms in the group of non-infectious choroiditis entities.

## 1. Introduction

Imaging exploration of the choroidal compartment used to be limited for inflammatory conditions before appropriate technologies became available. Echography using B-scan ultrasonography was one modality used to image inflammation of the choroid giving relatively useful information in the past on the structure, although it was rather rough, lacking the needed precision for fine analysis. Moreover, it gave only morphological information. It is not used any longer for choroiditis and is not part of the indications recommended in modern textbooks [1]. Fluorescein angiography (FA), principally offering information on the surface of the fundus (retina and optic disc), gives dynamic information but is not adapted for the choroid as the retinal pigmentary epithelium (RPE) blocks most of the rays in the visible spectrum of light, which is used in FA. It can therefore not be used to explore the choroid, except (1) in the first 40–60 s of angiography after injection of the fluorescein dye when the amount of choroidal fluorescein is very high, (2) when the RPE is missing (window-effect), or (3) when there is chorioretinal atrophy [2].

The situation changed in the early and mid-1990s when indocyanine green angiography (ICGA) became available for clinical use [3]. Thanks to ICGA, the choroid could be imaged as the rays were in the near infrared spectrum and could “see” through the RPE, allowing to analyse the choroidal fluorescence. Moreover, as for FA, ICGA provided dynamic information on the circulation of choroidal (and retinal) vessels and on the evolution of the choroidal ICG fluorescence during the angiographic phases. ICGA brought especially crucial progress in choroidal inflammatory diseases [4,5]. For the first time, the different levels of the choroid could be analysed with precision, allowing to classify and better understand the disease mechanisms of non-infectious and/or immune-mediated choroiditis [4,5,6,7].

Further progress in the appraisal of the choroid was achieved with an application of optical coherence tomography (OCT), namely enhanced depth imaging OCT (EDI-OCT), which allowed to measure choroidal thickness [8]. Although not as essential as ICGA, EDI-OCT was a useful adjunct for the investigation of choroidal inflammation [9]. More recently, OCT angiography (OCTA) also contributed to image the inner choroid at the level of the choriocapillaris [10,11], but is ill-suited for more deeper parts of the choroid, although studies with questionable practical implications have been published [12]. The drawbacks of these OCT methods reside in the fact that they only account for pathology in the posterior pole of the fundus [13]. It is the global pan-fundal imaging obtained with ICGA that lead to the classification of non-infectious choroiditis.

## 2. Indocyanine Green Angiographic (ICGA) Principles and Signs

### 2.1. Principles of ICGA

Indocyanine green angiography (ICGA) is characterised by two particular properties of the ICG molecule used in this procedure: (1) fluorescence at around 830 nanometres (nm) in the near infrared spectrum of light and (2) macromolecular behaviour [14].

#### 2.1.1. Fluorescence Characteristics of the ICG Molecule

**Figure 1:**
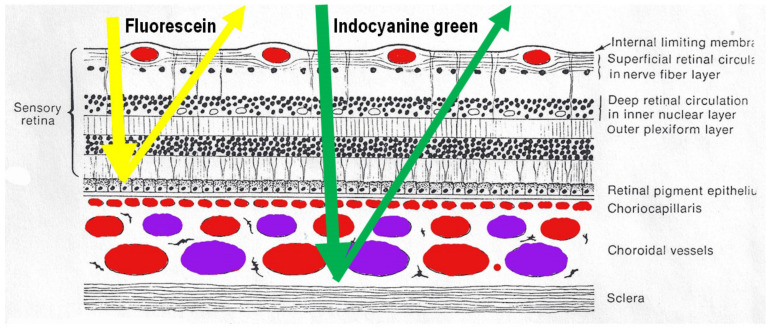
Difference between fluorescein (FA) and indocyanine green angiography (ICGA). FA is only able to analyse the fluorescence coming from the retina as the retinal pigmentary epithelium is blocking visible light fluorescence (yellow arrow), while ICGA is able to analyse retinal and choroidal near-infrared fluorescence (green arrow).

After intravenous injection of the ICG dye, fluorescence is obtained by shining a light beam inside the eye towards the fundus. Maximum absorption of the molecule occurs at around 800 nm, followed by fluorescence emission at around 830 nm. These infrared frequencies penetrate and reach the choroid through the RPE and conversely fluorescence produced by the ICG molecule can be detected through the RPE by appropriate angiography cameras. Fluorescein sodium produces fluorescence when excited by light frequencies in the visible light spectrum, which are stopped by the RPE. Therefore, FA cannot give information on the choroidal structures, while ICGA is detecting fluorescence coming from the choroid. (Figure 1) Some authors started to call this phenomenon cyanescence, which is a useless terminology, as the basic optical mechanism is fluorescence in both situations.

#### 2.1.2. Macromolecular Behaviour of the ICG Molecule

**Figure 2:**
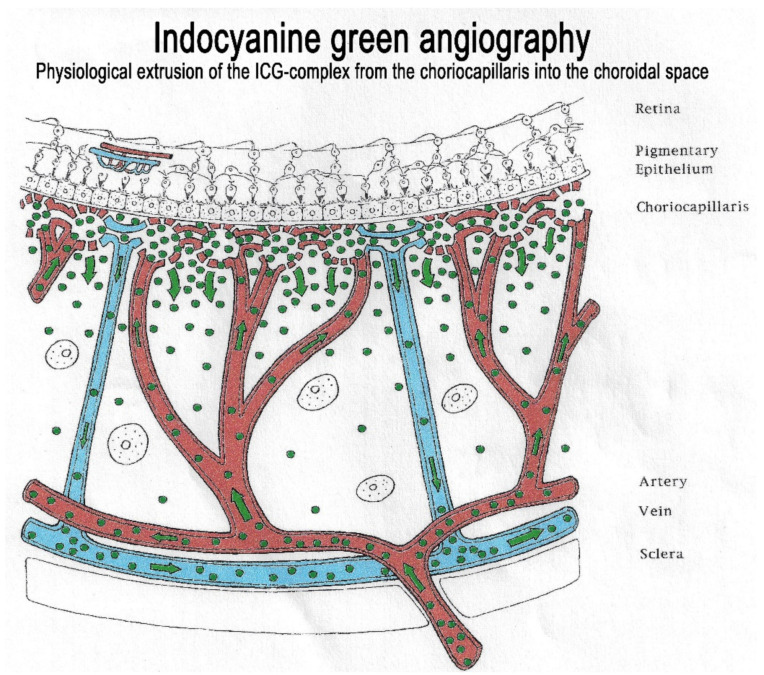
Indocyanine green angiography (ICGA). The ICG-protein complex remains intravascular in retinal vessels rendered impermeable by tight-junctions and in the large choroidal vessels but extrudes freely from the fenestrated choriocapillaris and impregnates progressively the choroidal stroma. It is these intermediate and late angiographic phases that are important for the evaluation of choroiditis.

The second crucial characteristic of the ICG molecule is that it is bound up to 98% to large plasma proteins (80% to globulins and 20% to alpha-lipoproteins and albumins). ICG has a molecular weight of 775 Daltons (d), not much more than the fluorescein molecule (332 d). The difference, however, lies in the fact that, being tightly bound to large proteins, it forms a large molecular complex with a molecular weight of over 60 to 80,000 d. This means that ICG will remain within the vessels of the retina, which have tight junctions. In the choroid also, ICG will remain within large vessels. However, this large molecular complex will escape from the fenestrated choriocapillaris and will shed into the choroidal space and remain trapped as wash-out will be slow because of the size of the molecular complex. (Figure 2) Consequently, analysis of ICGA fluorescence is more focussed on the choriocapillaris flow and the subsequent impregnation of the choroidal space by the ICG molecular complex than the intravascular fluorescence. It is by analysing the pattern of Impregnation of the choroidal space and its disturbance that ICGA made it possible to classify choroiditis and its different mechanisms.

### 2.2. Interpretation of ICGA in Non-Infectious Choroiditis According to Angiographic Pattern

**Figure 3:**
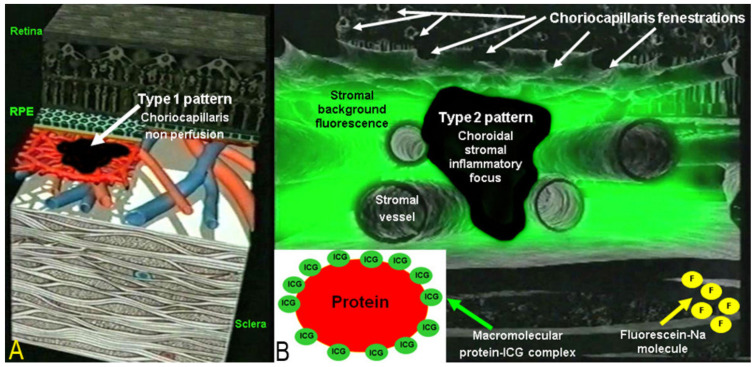
Indocyanine green angiography (ICGA) patterns in non-infectious choroiditis. Two main patterns have been identified in choroiditis. Pattern 1 is seen in inflammatory choriocapillaris non-perfusion (**A**). Pattern 2 is found in stromal inflammation when foci develop and impair the diffusion of the ICG-protein complex shedding from the fenestrate choriocapillaris (**B**). The ICG-protein complex is shown in the insert, which is much larger than the fluorescein molecule with a slow wash-out from the choroid.

Two main patterns of ICGA signs in choroiditis have been identified, as shown on Figure 3.

**Figure 4:**
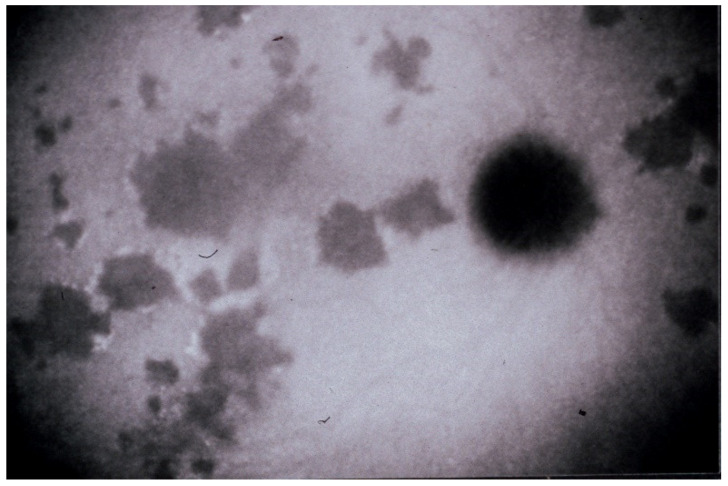
Indocyanine green angiography (ICGA), type 1 pattern: choriocapillaris non-perfusion. Typical geographic areas of dark non-fluorescence in case of acute posterior multifocal placoid pigment epitheliopathy or acute multifocal ischaemic choriocapillaritis (APMPPE/AMIC).

(1) Inflammatory choriocapillaris non-perfusion or hypoperfusion, represented on the Figure 3A appears as irregular geographic areas of hypofluorescence or absence of fluorescence (Figure 4). This pattern is found in primary or secondary choriocapillaritis or inflammatory choriocapillaropathies.

A useful complimentary imaging modality for choriocapillaritis is fundus autofluorescence (FAF,) in particular, blue-light fundus autofluorescence (BAF). Fundus BAF hyperautofluorescence co-localises with ICG hypofluorescence. This is explained by the fact that choriocapillaris non-perfusion causes ischaemic damage and loss of the photoreceptor outer segments. The loss of this screen due to the photopigments causes unmasking of normal underlying RPE autofluorescence, thus producing BAF hyperautofluorescence [15]. This imaging modality is very useful, as the evolution of the choriocapillaritis can be followed without the need to repeat ICGA [16].

**Figure 5:**
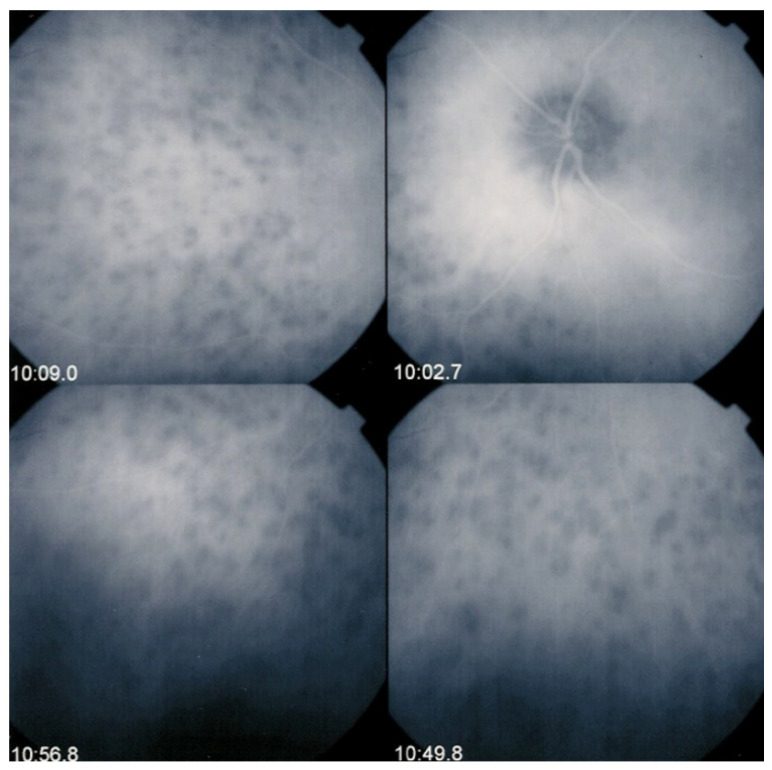
Indocyanine green angiography (ICGA), type 2 pattern found in stromal choroiditis. Typical round HDDs of similar size, evenly distributed caused by the presence of inflammatory foci that impair the diffusion of the ICG dye showing granulomas in negative in case of Vogt-Koyanagi-Harada disease (VKH).

**Figure 6:**
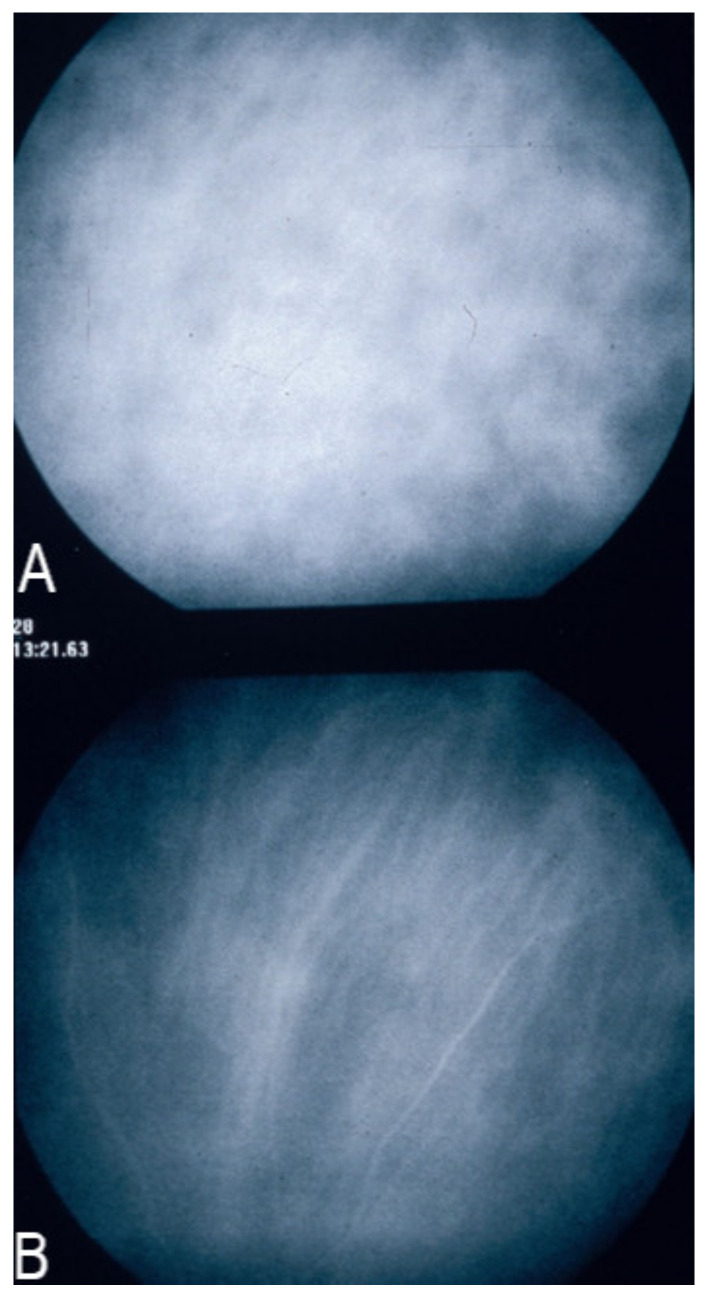
Indocyanine green angiography (ICGA), type 2 pattern found in stromal choroiditis. (**A**): Typical round HDDs of similar size evenly distributed caused by the presence of inflammatory foci that impair the diffusion of the ICG dye showing granulomas in negative in case of Vogt-Koyanagi-Harada (VKH) disease. Choroidal vessels are no more distinct and appear fuzzy with diffuse hyperfluorescence hiding the HDDs. (**B**): After three days of intravenous corticosteroids, the choroidal vessels are again distinctly visible, and HDDs have partially resolved.

(2) On the other hand, stromal choroiditis produces type 2 ICGA pattern characterised by round, often numerous and evenly distributed hypofluorescent dark dots (HDDs) (Figure 5) generated by stromal inflammatory foci that impair the diffusion of the ICG-protein complex, as shown in Figure 3B. If the foci do not occupy the full-thickness of the choroid, they can fade and become isofluorescent in late angiographic frames. Apart from HDDs, ICGA shows additional leakage from larger choroidal vessels that are less distinct and appear fuzzy. (Figure 6). The whole set of angiographic signs that can be identified in stromal choroiditis is listed on Table 1. A grading of these signs allowed to establish a precise quantitative angiographic score [17].

Unfortunately, all choroiditis conditions have been grouped under the term of white dot syndromes in the past, albeit the fact that disease mechanisms clearly differ. Such a terminology is therefore inappropriate, and these diseases should be classified into choriocapillaritis entities/inflammatory choriocapillaropathies on one side and stromal choroiditis entities on the other side, according to the disease mechanism uncovered by ICGA [18,19,20,21].

## 3. ICGA-Derived Classification and Clinicopathological Mechanisms in Non-Infectious Choroiditis and Specific Entities

### 3.1. Choriocapillaritis Entities or Inflammatory Choriocapillaropathies

Choriocapillaritis entities are classically subdivided into primary choriocapillaritis [19], including conditions for which the trigger is not known and supposed to be of viral origin, as many of these cases report flu-like symptoms preceding the choriocapillaritis. The most well-known conditions in this group are Multiple Evanescent White Dot Syndrome (MEWDS), Acute Posterior Multifocal Placoid Pigment Epitheliopathy (APMPPE) or Acute Multifocal Ischaemic Choriocapillaritis (AMIC), Idiopathic Multifocal Choroiditis (MFC), and Serpiginous Choroiditis (SC). In the group of secondary inflammatory choriocapillaropathies, the trigger for the development of choriocapillaritis is known such as in Acute Syphilitic Posterior Placoid Chorioretinitis (ASPPC) [22,23] or in tuberculosis-related serpiginous choroiditis [24].

#### 3.1.1. Primary Inflammatory Choriocapillaropathies (PICCPs)

The type and extent of lesions in primary choriocapillaritis seems to depend on the level of vascular involvement determining a spectrum of disease severity from benign and reversible involvement in MEWDS to severe and irreversible lesions in MFC and SC. Choriocapillaritis cases may correspond to one of the four well-known described syndromes, including MEWDS, APMPPE/AMIC, MFC, and SC or may correspond to mixed and overlapping intermediary forms or cannot be classified into the precisely described clinical entities.

The purpose in this article on classification is not to give an exhaustive description of the different entities, which will be dealt individually in other articles of this special issue.

##### Multiple Evanescent White Dot Syndrome/Acute Idiopathic Blind Spot Enlargement (MEWDS/AIBSE)

MEWDS is a well-characterised choriocapillaritis at the benign end of the spectrum of inflammatory choriocapillaropathies [25]. It was precisely described by Lee Jampol et al. in 1984 [26]. When first described, it was still thought to be a retinal pigment epithelial disease [27]. With the availability of ICGA it became clear that MEWDS was a disease of the choriocapillaris [28,29,30].

**Figure 7:**
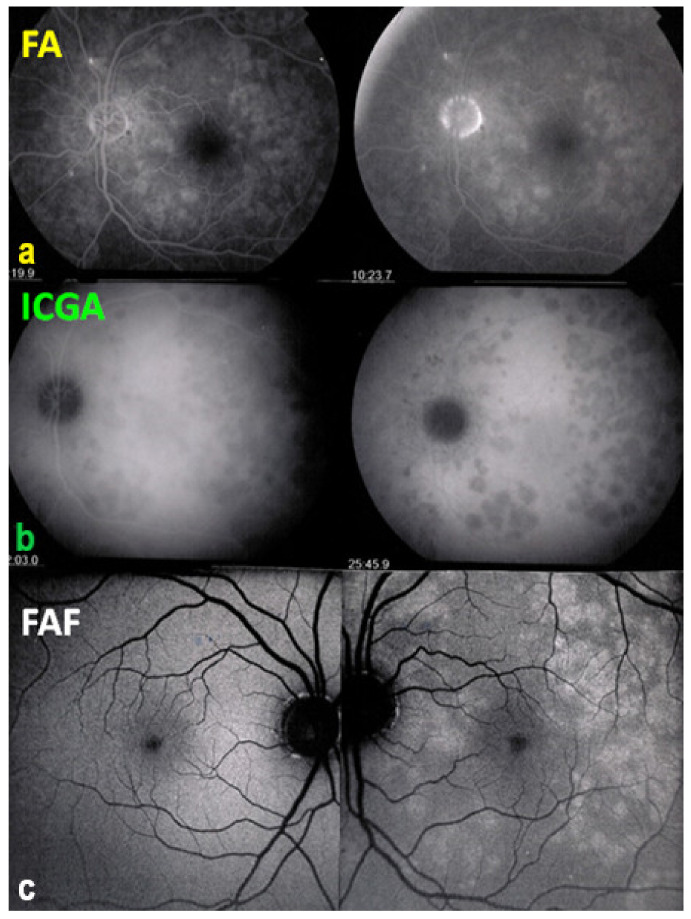
Multimodal imaging of a case of MEWDS. FA (**a**) shows faint FA hyperfluorescence probably caused by outer retinal ischaemia. ICGA (**b**) shows extensive zones of hypofluorescence due to choriocapillaris non-perfusion. FAF (**c**) shows extensive BAF hyperautofluorescence that co-localise with ICGA hypofluorescent areas and is produced by the loss of the photopigment screen unmasking the normal underlying RPE autofluorescence.

**Figure 8:**
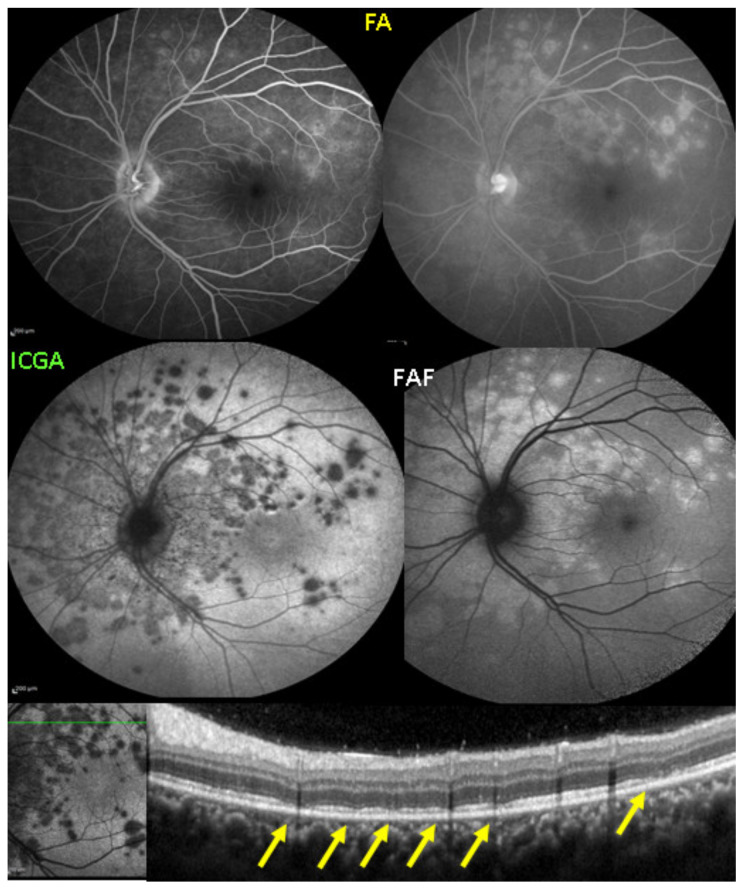
SD-OCT in a case of MEWDS. Loss of ellipsoid zone (photoreceptor outer segments (arrows) corresponding to the area where BAF hyperautofluorescence and ICGA hypofluorescence are maximal.

The disease usually affects young persons with a predilection for women and is unilateral. Flu-like symptoms precede the ocular involvement in up to 50% of cases. It has also been associated with influenza and other vaccinations [31]. Symptoms typically consist in visual impairment with visual loss that can be severe, subjective scotomas, and photopsias. The clinical signs include faint discoloured dots along the temporal arcades, in the mid-periphery and the posterior pole that can have disappeared when the patient finally consults. What remains a little longer is a granular aspect of the macula [32]. Functionally, visual loss can be moderate to severe as is the case for visual field changes, which can be discreet to large central scotomas. The most important investigation used to be ICGA which was essential for the diagnosis when clinical signs had already resolved. However, nowadays multimodal examination is performed including FA, BAF, ICGA, and OCT (Table 2) (Figure 7 and Figure 8).

Recently, the primary role of choriocapillaris inflammatory non-perfusion has been put in doubt for MEWDS [33]. The authors based their hypothetical theory on the fact the choriocapillaris seems to be intact by OCTA. Nevertheless, in all cases where ICGA was performed, it showed hypofluorescent areas representing hypo or non-perfusion of end-capillaries.

OCTA is unable to show end-capillary circulation. Hence, its absence or presence, in this low-flow mode is not “seen” by OCTA as this modality needs sufficient flow to identify the presence or absence of vessels, which is, conversely, clearly apparent (or not) on ICGA [34].

In most cases, MEWDS does not need any treatment and resolution occurs in 8 to 10 weeks and once the diagnosis is made, it is important to reassure the patient.

AIBSE (acute idiopathic blind spot enlargement) was described in 1988 in a report including seven patients that presented a peripapillary scotoma producing symptomatic enlargement of the blind spot objectively identified by visual field testing. They were all young patients aged from 25 to 39 years with a 5/2 female predominance [35]. Most probably AIBSE and MEWDS are the same disease with the exception of the usual fundus findings that were not found in this and other reports on AIBSE. If ICGA had been available and performed by the authors of this and subsequent reports, it is probable that AIBSE would never have been described as a separate entity, as ICGA is presently the method of choice for making the diagnosis of atypical MEWDS in patients consulting at a later stage of the disease or presenting subclinical disease [36]. The reports including ICGA investigation in their work show that visual field alterations are related to peripapillary hypofluorescence, indicating choriocapillaris non-perfusion as the physiopathogenic process at the origin of blind spot enlargement [29,36,37].

##### Acute Posterior Multifocal Placoid Pigment Epitheliopathy/Acute Multifocal Ischaemic Choriocapillaritis (APMPPE/AMIC)

The level of involvement of choriocapillaris non-perfusion in APMPPE/AMIC touches larger portions and choriocapillaris drop-out is detected by OCTA and no one will contest that the primary mechanism in APMPPE is choriocapillaritis. Like for MEWDS, the disease origin was first attributed to the RPE by Gass as ICGA was not yet available [38]. Nevertheless, Deutmann did not need ICGA to understand the clinicopathological mechanism of the disease. By observing the early frames of FA, he understood that the choriocapillaris was the culprit and he named the disease in a justified manner AMIC (Acute Multifocal Ischaemic Choriocapillaritis) [39,40,41]. Very often during meetings, these two exceptional clinicians clashed over this issue. Once ICGA became available, the primary role of the choriocapillaris was clearly established [42].

APMPPE/AMIC, unlike MEWDS, is a bilateral disease, although involvement may be asymmetric and there may be a delay before the second eye is involved [43]. APMPPE/AMIC affects young patients and, as for other PICCPs, it is preceded in up to 50% of cases by a flu-like episode or other infectious episodes such as mumps or a streptococcal infection or in connection with a vaccination [44,45,46]. It is important to know that in rare cases, APMPPE/AMIC can be associated with a cerebral vasculitis [47,48]. Significant anterior uveitis can occur in rare instances.

Like in other PICCPs, patients complain of visual disturbance including drop of visual acuity, subjective scotomas, and photopsias. Decrease of visual acuity and visual field changes depend on the severity of involvement but more importantly on the localisation of the area involved.

**Figure 9:**
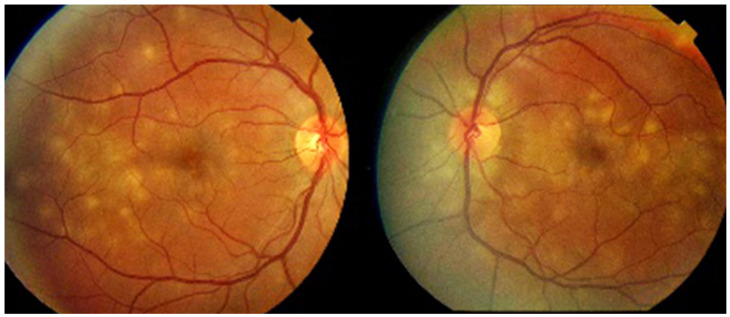
Numerous bilateral discolored plaques in a case of APMPPE/AMIC typical bilateral posterior disposition of placoid lesions.

Investigation of APMPPE/AMIC has become much more precise with the availability of multimodal imaging (Table 3). Fundus examination shows multiple pale discolored plaques (Figure 9).

**Figure 10:**
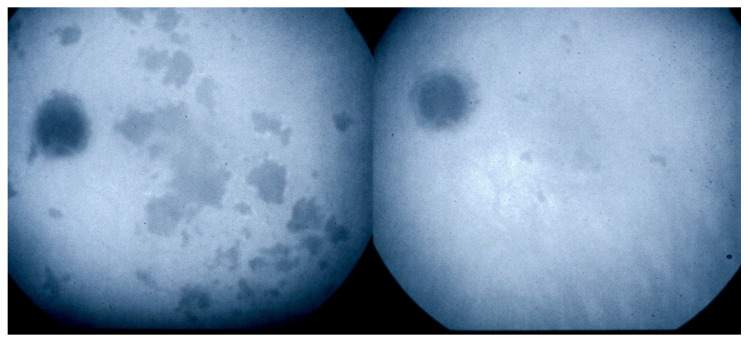
APMPPE/AMIC. Geographic areas of ICGA hypofluorescence.

**Figure 11:**
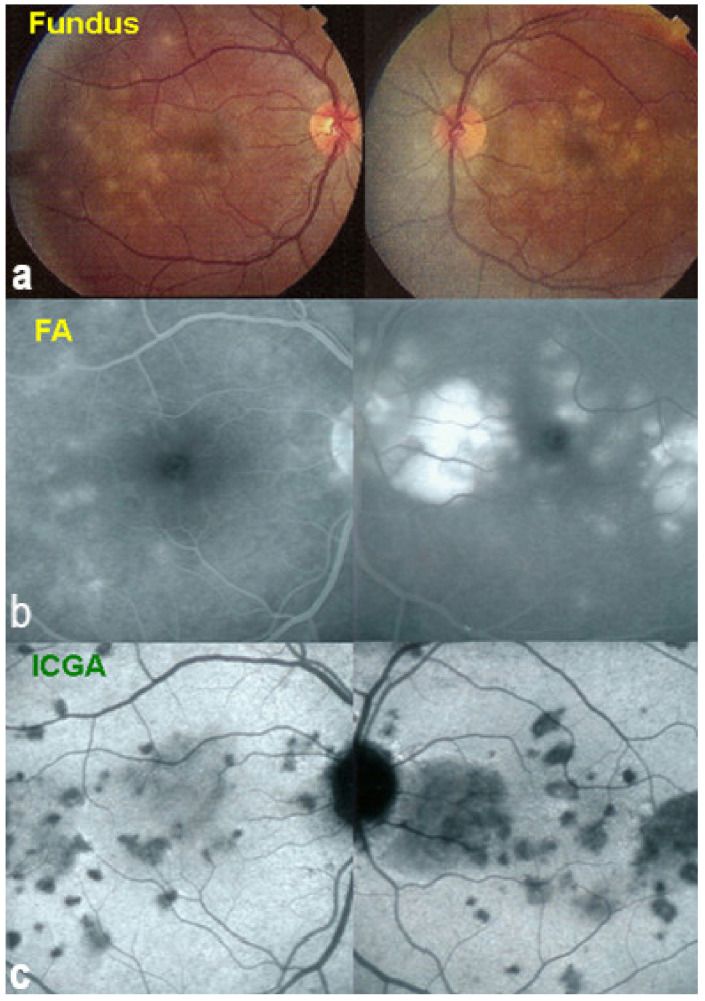
Case of APMPPE/AMIC. Areas of fundus discoloration due to pooling of fluid under the retina (**a**). FA shows pooling of liquid under the retina (**b**). ICGA shows choroidal non-perfusion (**c**).

**Figure 12:**
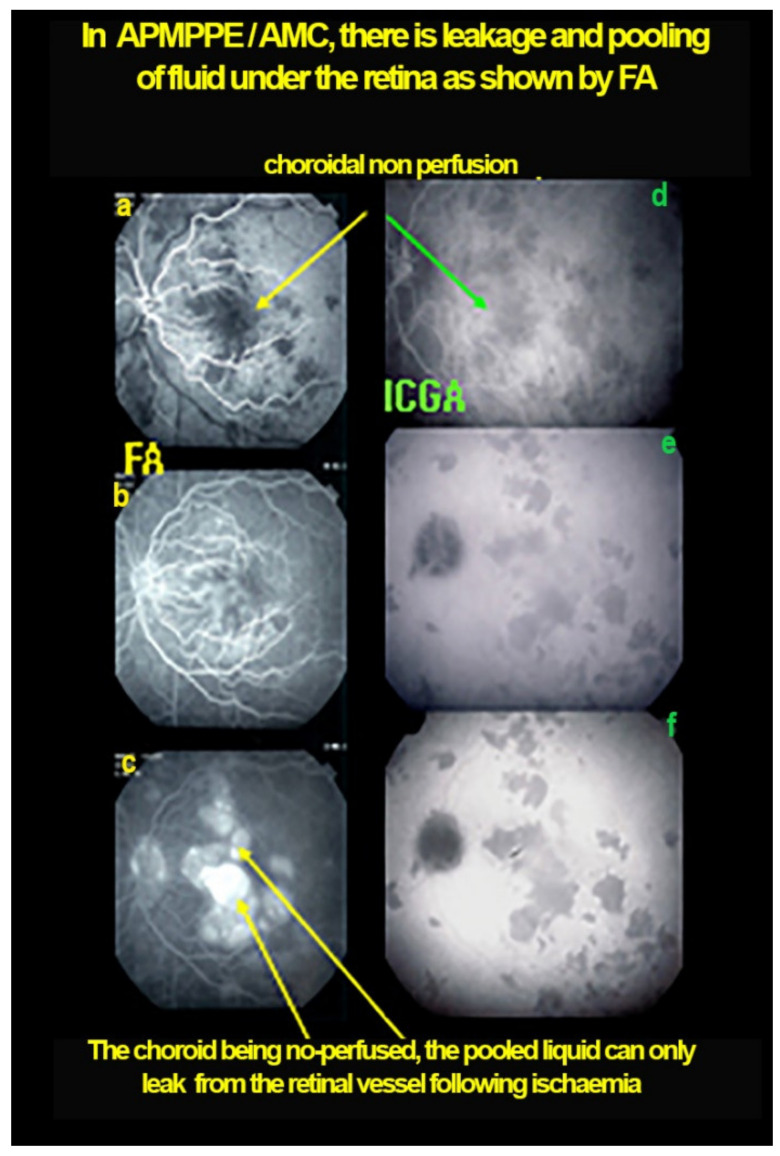
Case of APMPPE/AMIC: explanation of FA pooling (FA, left column, ICGA, right column) Choroidal (choriocapillaris) non-perfusion causes retinal ischaemia with retinal vessel increased permeability causing leakage and pooling under the retina. Early frames (**a**,**d**), mid-phase (**b**,**e**) and late phase (**c**,**f**).

As the affected areas involved are significantly larger than in MEWDS, ICGA hypofluorescence is much more pronounced and widespread (Figure 10). The ischaemic consequences on the retina are also much more important than in MEWDS where ischaemia-induced FA hyperfluorescence remains usually faint. In contrast, in APMPPE/AMIC, FA shows early hypofluorescence due to choriocapillaris non-perfusion. In the later phase of FA, pronounced hyperfluorescence and even pooling occurs due to massively ischaemia-induced increased permeability of both the outer and inner retinal vascular plexuses, which correspond to the yellow discoloured plaques seen on fundus examination (Figure 9). Exudation and pooling in late FA frames can only come from retinal vessels in response to outer retinal ischaemia, as the underlying choroid is non-perfused (Figure 11 and Figure 12). FAF shows hyperautofluorescence in moderately involved areas (loss of photoreceptor outer segments) but hypoautofluorescence in areas with severe vascular drop out. SD-OCT shows loss of photoreceptor outer segments in moderately involved areas. OCTA shows vascular drop-out.

Although reversibility of lesions without treatment is usually reported, close ICGA follow-up should be practiced and there should be no hesitation to initiate systemic corticosteroid treatment if imaging monitoring is not showing improvement.

**Table 3:** Multimodal imaging of APMPPE/AMIC lesions and clinicopathological explanation.

BAF	Hyperautofluorescence (increased exposure of RPE lipofuscin following loss of outer photoreceptor segments)
	Hypoautofluorescence in severely affected areas
ICGA	Extended hypofluorescent areas (choriocapillaris ± larger vessel non perfusion) (Figure 11)
OCT	Loss of photoreceptor outer segments ± chorioretinal atrophic areas
OCTA	Choriocapillaris drop-out
FA	Early hypofluo in diseased areas (non-perfusion) then diffuse hyperfluo/pooling (due to retinal ischaemia) (Figure 12)

BAF = Blue-light fundus autofluorescence; ICGA = indocyanine green angiography; SD-OCT = spectral domain optical coherence tomography; OCTA = OCT angiography; FA = fluorescein angiography.

##### Idiopathic Multifocal Choroiditis (MFC)

**Figure 13:**
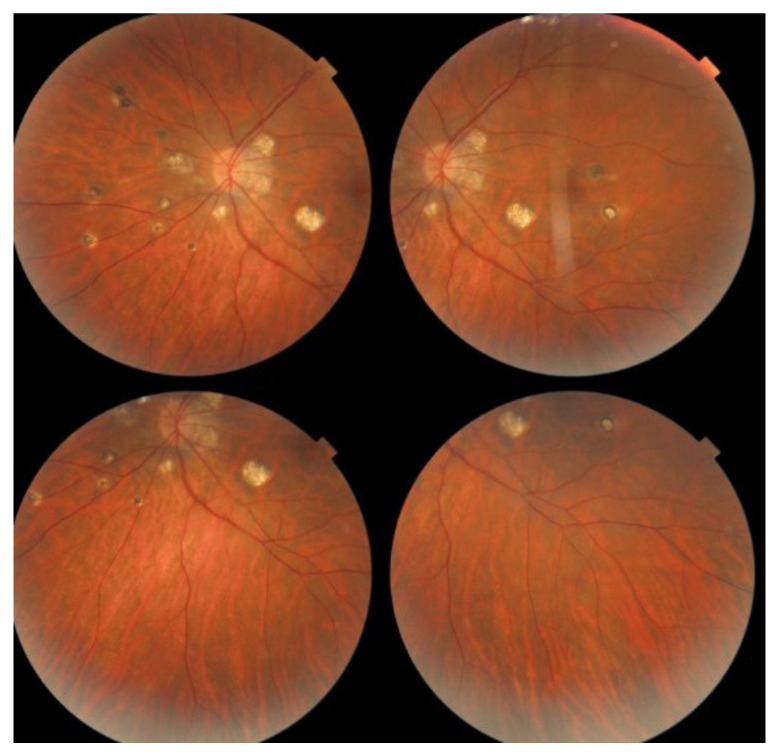
Idiopathic Multifocal choroiditis (MFC), fundus image. Multiple yellow foci typically seen in MFC (healed stage).

MFC is situated towards the more severe end of the spectrum of PICCPs. In 1973, Nozik and Dorsch described an entity which they called multifocal uveitis and panuveitis [49]. In 1984, Dreyer and Gass published a report entitled “Multifocal choroiditis and panuveitis, a syndrome that mimicked ocular histoplasmosis syndrome”, reporting 28 additional cases [50]. The panuveitis part in most cases is very minimal and mostly limited to cells in the posterior vitreous. Therefore, the name that should more appropriately be used is just multifocal choroiditis (MFC), as panuveitis is not in the foreground for most cases seen nowadays. Since the editorial published in the journal Retina in 2013, the term of idiopathic multifocal choroiditis (MFC) has been put forward and regroups many of the cases described separately, including punctate inner choroidopathy (PIC) and other diversely described sub-entities such as multifocal inner choroiditis, recurrent multifocal choroiditis, etc. [51]. The cases described as “pseudo or presumed ocular histoplasmosis syndrome (POHS)” in patients with negative circulating antibodies to histoplasma capsulatum and a negative hypersensitivity skin test and coming from non-endemic areas for histoplasmosis are now also assimilated to idiopathic multifocal choroiditis, POHS being a non-entity [19,51]. The characteristics of all the subtypes of MFC are the numerous small randomly distributed chorio-retinal scars (Figure 13) and the recurrent behaviour of the disease as well as the propensity to develop secondary neovascular membranes, which is much more frequent than in all other PICCPs. Multifocal choroiditis occurs in the same age groups as other PICCPs, namely in young to middle aged adults with myopic women being predominantly affected [52]. Lesions tend to leave scars.

The symptoms that connect multifocal choroiditis to all other PICCPs are the photopsias and subjective scotomas. MFC is usually unilateral but can sequentially affect both eyes during recurrences. Visual loss depends on the localisation of the lesions and can be severe when lesions are close to the fovea. Visual field testing objectively identifies the scotomas the patients report and that are localised to the areas of fundus involvement. Slight vitreous and anterior inflammation is rarely seen.

**Figure 14:**
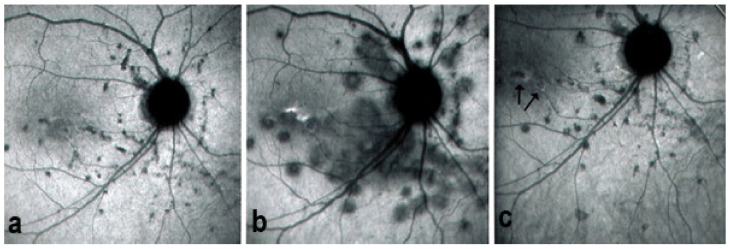
MFC. ICGA time sequence. (**a**): shows quiet stage. Middle picture (**b**) shows reactivation of MFC eight months later. (**c**): shows healed stage of choriocapillaritis six months later after systemic corticosteroid therapy.

On fundus examination, the typical lesions are small randomly distributed choroidal mostly atrophic yellow-white foci with pigment spots (Figure 13) that sometimes can become adjacent to each other and form a ribbon of pearls. These lesions involve the posterior pole, around the disc, as well as the mid-periphery. In the active phases of disease, new lesions are not always visible and can be very discreet on FA, whereas ICGA is the most sensitive method to detect new lesions, as is the case in MEWDS [30,37,53,54,55]. (Figure 14) One particular feature of multifocal choroiditis is the high proportion of choroidal neovascular membranes complicating the disease, occurring in as much as one-third of cases.

**Figure 15:**
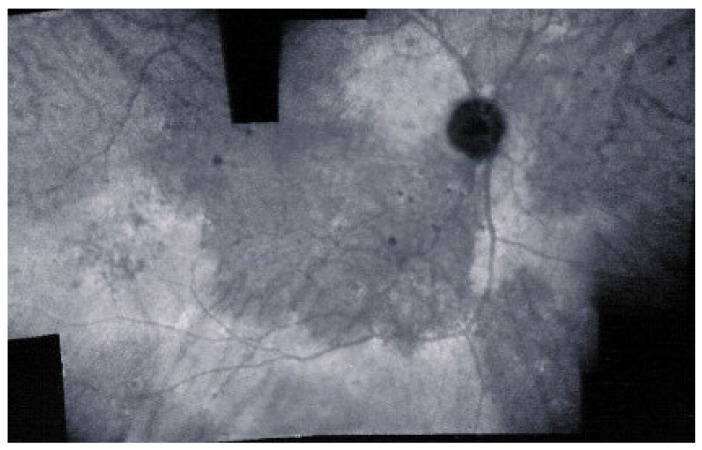
Vast areas of ICGA hypofluorescence indicating choriocapillaris hypo or nonperfusion surrounding the disc. These areas indicate that in some cases, the inflammatory process going on sub-clinically is involving vast areas of choriocapillaris perfusion problems and can be a trigger for the development of CNVs.

As for all PICCPs, multimodal imaging is best accounting for MFC features (Table 4)**.** On ICGA, the first set of signs identifies old scarred chorioretinal lesions and consists of hypofluorescent areas persisting up to the late angiographic phase, distributed at random in the fundus, corresponding to late hyperfluorescence on FA, typical for chorioretinal atrophy from scars of previous inflammatory episodes seen on colour fundus photos. The second set of signs can be seen in addition to the previously described signs when choroiditis recurs or can be seen in the absence of scars when it is the first episode. The signs consist of hypofluorescent areas, either silent on fluorescein angiography or hyperfluorescent in the late phase and usually not visible on fundus examination, representing areas of new inflammatory involvement (Figure 14). As in many PICCPs, some cases may present peripapillary hypofluorescence, translating functionally into an enlarged blind spot [18,36,54,55] (Figure 15). The hypofluorescent areas can completely regress if inflammation suppressive treatment is started promptly. In a substantial proportion of cases, the extent of ICGA hypofluorescence reflecting choriocapillaris hypoperfusion or nonperfusion is far more widespread than visible lesions let suspect, showing widespread areas of late occult hypofluorescence with absolutely no signs visible on fundus examination or on fluorescein angiography (Figure 15).

**Figure 16:**
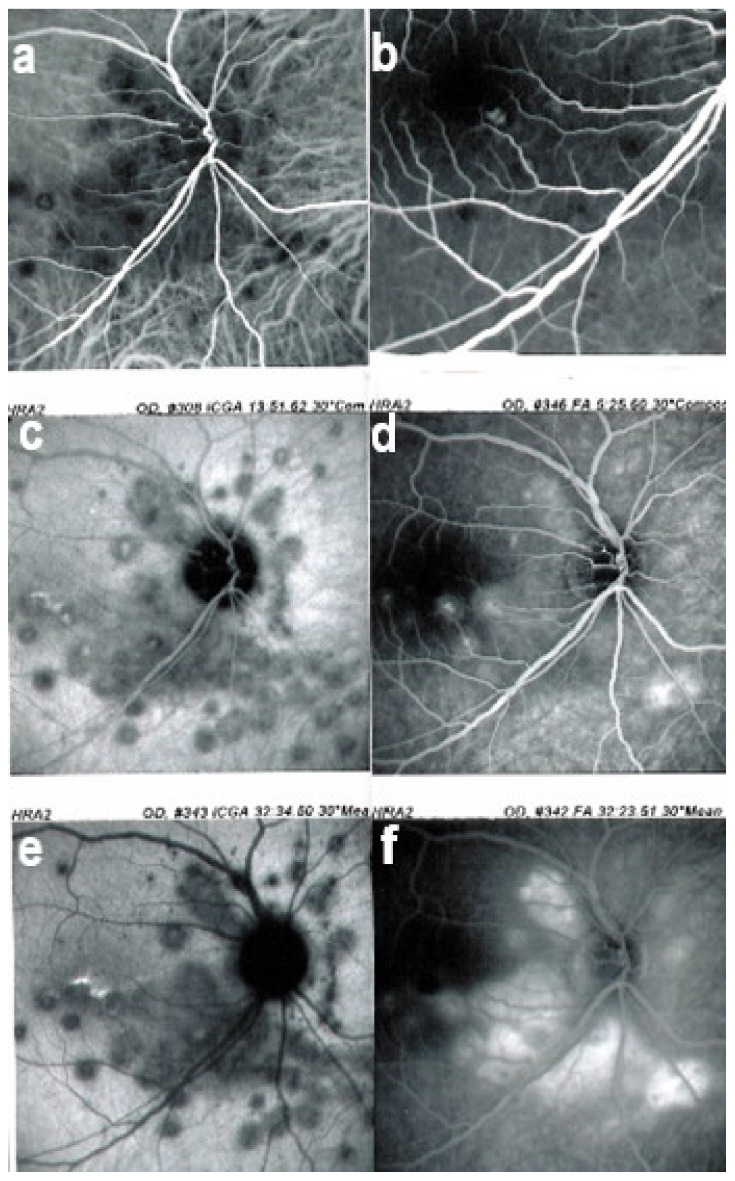
MFC. Parallel time sequence of ICGA (**a**,**c**,**e**) and FA (**b**,**d**,**f**) during recurrence. Top pair (**a**,**b**) show early ICGA and FA frames, showing extended hypofluorescent area on ICGA, while FA is practically normal. Middle pair (**c**,**d**) of images show intermediate phase of ICGA and FA (about 10 min) with more extended and well-visible zones of hypofluorescent choriocapillaris nonperfusion on ICGA, while FA shows leakage and staining. Bottom images (**e**,**f**) corresponding to late ICGA hypofluorescent areas causing retinal ischaemia and reactional retinal vessel hyperpermeability following the same mechanism as in APMPPE/AMIC.

FA shows mainly signs of chorioretinal scaring associating by window effect (late sclera hyperfluorescence due to staining) to masking effects where there is pigment clumping. In the active phase, FA may show faint late hyperfluorescence in areas corresponding to ICGA, hypofluorescent dark areas corresponding to new lesions. In case of severe hypoperfusion of the choriocapillaris, bright FA late hyperfluorescence (retinal and subretinal staining) can occur as for APMPPE/AMIC (Figure 16). The use of FA is, however, of little contribution to assess and follow active disease.

SD-OCT is very helpful as in all other PICCPs. In MFC, OCT pictures show that the degree of repercussion of choriocapillaris non-perfusion on the outer retina and even inner retina is much more pronounced in active phases of MFC when compared to MEWDS or APMPPE/AMIC. This seems to indicate that larger choroidal vessels seem to be involved, as suggested by the only histopathological report available [56]. It is understandable that OCT findings in MFC and APMPPE/AMIC are similar as it is the same mechanism of choriocapillaris closure ± larger vessels with more or less severe consequences on the retina.

FAF in MFC shows increased autofluorescence in those areas that have silent (meaning without FA signs) ICGA hypofluorescent lesions and hypoautofluorescence in the cicatricial areas. After inflammation suppressive treatment, hyperautofluorescence disappears in parallel with resolution of ICGA hypofluorescence. The areas showing hyperautofluorescence go beyond the ICGA hypofluorescent areas, indicating that inflammatory involvement go even beyond the areas detected by ICGA.

**Figure 17:**
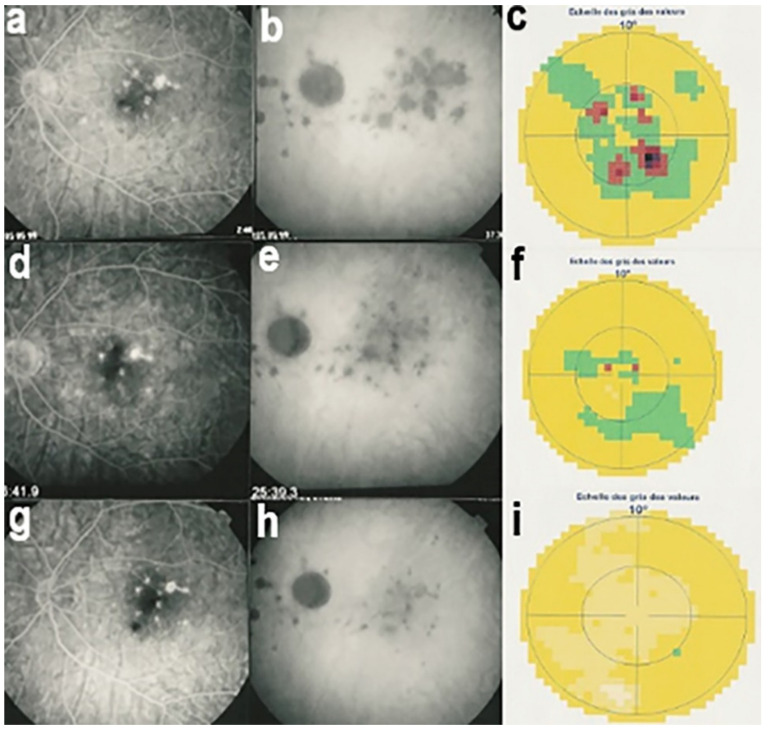
Correlation between FA, ICGA, and visual field during corticosteroid treatment of MFC. This myopic patient presented with left eye photopsias, left decrease of visual acuity (VA) and the presence of a subjective left scotoma. FA (**a**) only shows cicatricial FA lesions corresponding to cicatricial fundus lesions seen on fundus examination. ICGA (**b**) shows widespread hypofluorescent areas, indicating fresh choriocapillaris lesions with corresponding visual field defects (**c**). During corticosteroid therapy, practically no change is seen on FA (**d**,**g**), whereas ICGA signs of active disease progressively resolve (**e**,**h**) together with visual field improvement (**f**,**i**).

Visual field testing can show small scotomas corresponding to the chorioretinal scars. In the active phase of disease, however, scotomas are larger and correspond to choriocapillaris non-perfusion shown on ICGA. Visual field recovery is well correlated with the regression of ICGA hypofluorescent areas that occurs following introduction of inflammation suppressive treatment [30] (Figure 17). On the other hand, recovery is not well correlated to FA.

**Figure 18:**
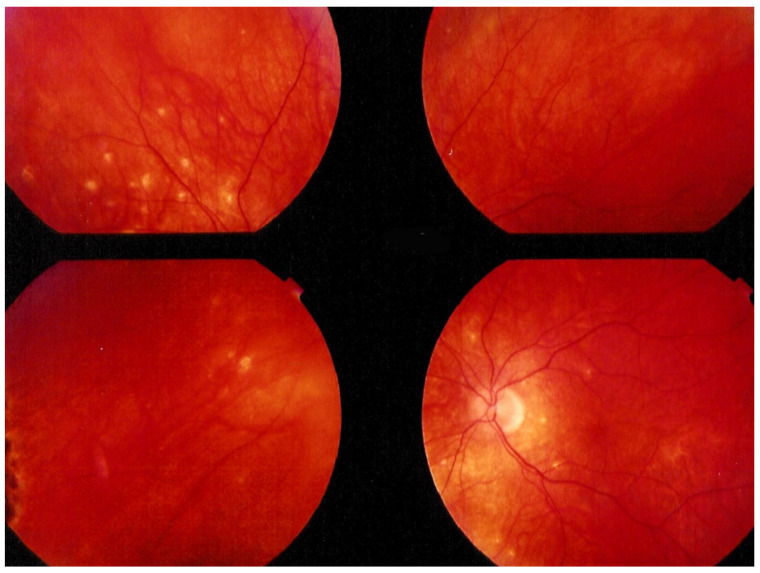
Fundus pictures of MFC showing retinochoroidal scars at the end of a recurrent episode followed by FA (Figure 19) ICGA (Figure 20) and by FAF (Figure 21).

**Figure 19:**
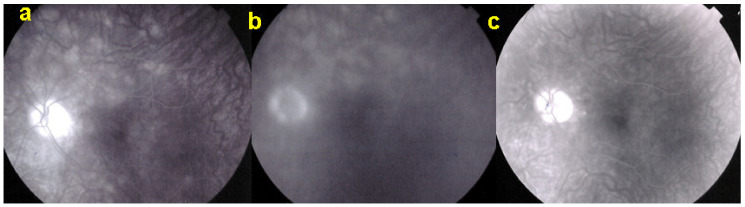
FA pictures of MFC at presentation (**a**), after 1 month (**b**) and 4 months (**c**). Patchy hyperfluorescent areas are seen at presentation around optic disc and along temporal superior arcade, indicating staining of retina caused by ischaemia of outer retina (**a**), still present at 1 month (**b**), almost resolved after 4 months (**c**).

**Figure 20:**
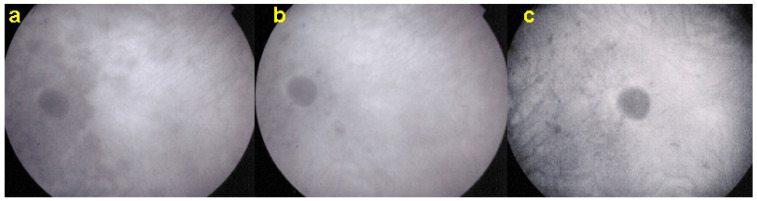
ICGA pictures of MFC at presentation (**a**), after 1 month (**b**) and 4 months (**c**). Peripapillary confluent hypofluorescence and scattered hypofluorescent areas along superior temporal arcade at presentation (**a**), which almost completely resolved after one month (**b**). However, at 4 months, (**c**) hypofluorescent areas are still seen.

**Figure 21:**
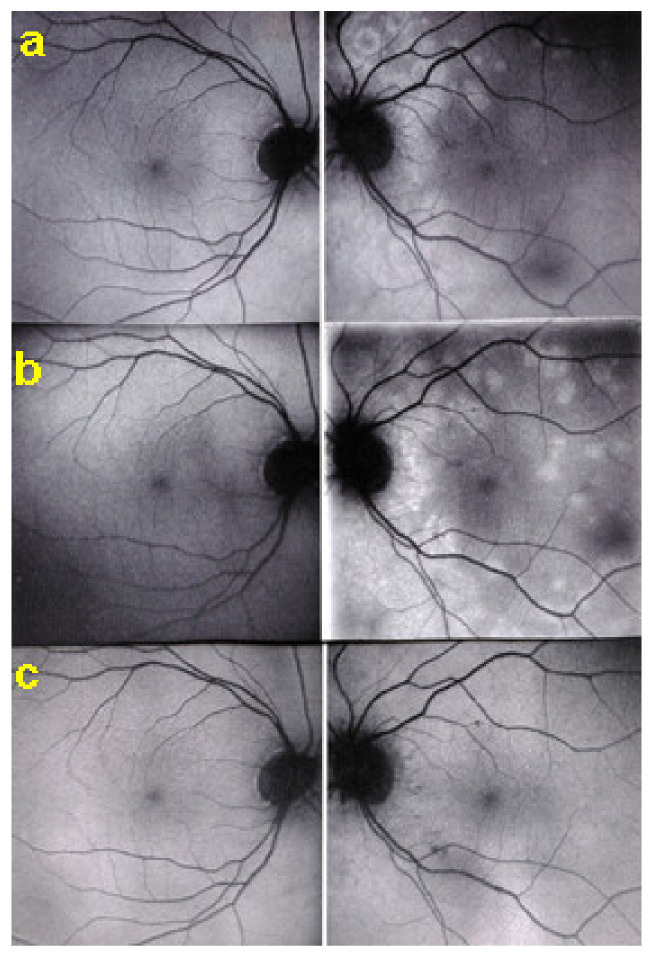
BAF pictures of MFC at presentation (**a**), after 1 month (**b**), and 4 months (**c**). On the right column of pictures (left eye), hyperautofluorescent areas corresponding to the FA and ICGA lesions can be seen at presentation (**a**) which, on the (**b**), have slightly progressed at one month with return to almost normal autofluorescence after 4 months (**c**). BAF delineates lesions even with more precision than ICGA.

**Figure 22:**
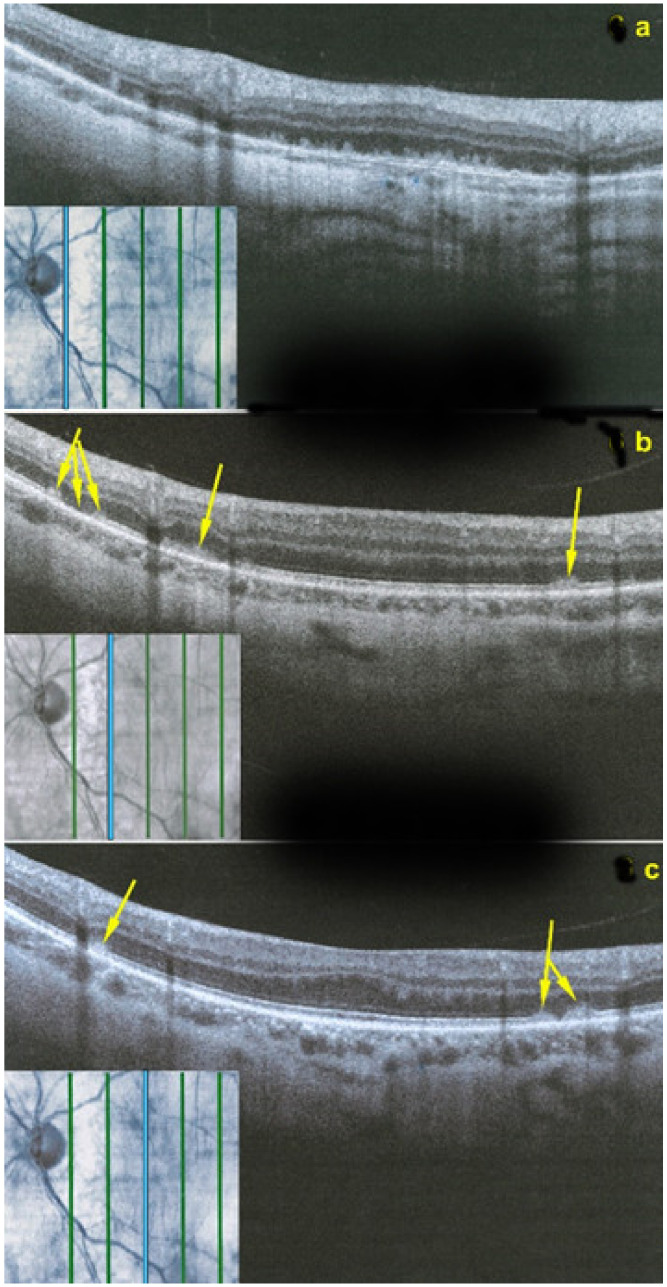
OCT scans of MFC at presentation through ICGA hypofluorescent area (**a**), at the border of ICGA hypofluorescent area (**b**,**c**). The top set of figures (**a**) represents a scan going through the ICGA hypofluorescent area (FAF hyperautofluorescent) showing damaged photoreceptor outer segments with clumps throughout the whole scan. (**b**,**c**) show scans at the border of the ICGA hypofluorescent (FAF hyperautofluorescent) area with only sectorial damage to the photoreceptor outer segments (arrows) and almost normal ellipsoid zone on the most distant scan from ICGA hypofluorescent (FAF hyperautofluorescent) area.

Although treatment is purely empirical, aggressive inflammation suppressive treatment associating corticosteroids and non-steroidal immunosuppressive agents can be recommended in case of newly diagnosed active disease or reactivation of MFC. Monitoring of therapy is best done by ICGA, which is the most sensitive modality to detect activity showing progression or regression of hypofluorescent areas [30]. BAF monitoring gives similar information for the follow-up of lesions and has the advantage of not being invasive, (Figure 18, Figure 19, Figure 20 and Figure 21) corresponding to loss of outer segments of photoreceptors visible on SD-OCT (Figure 22)

Sub-Tenon’s corticosteroid injections can be tried if disease or reactivation is unilateral. However, if no improvement is seen, dual systemic inflammation suppressive treatment is necessary, including steroidal and non-steroidal immunosuppression. In case of the presence of CNVs, intravitreal injections of anti-VEGF agents have to be administered.

##### Serpiginous Choroiditis (SC)

Serpiginous choroiditis (SC) is at the most severe end of the PICCPs. It is a progressive recurrent bilateral primary inflammatory choriocapillaropathy that leads to irreversible destruction of the chorio-retina if immunosuppressive therapy is not started promptly [57]. It is also called geographical or helicoid choroidopathy [58,59] and affects more elderly patients in contrast to the young healthy adult age group traditionally affected by the other PICCPs. It has to be distinguished from serpiginous-like or serpiginoïd choroiditis phenotypically very close to SC but occurring in patients that have been exposed to Mycobacterium Tuberculosis [60,61]. Therefore, this latter form is classified in the group of secondary inflammatory choriocapillaropathies and will be dealt later in this section.

**Figure 23:**
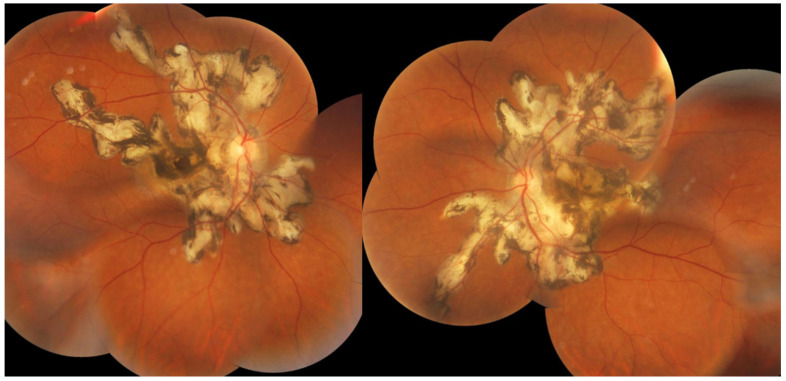
SC: serpiginous lesions on fundus photography. Bilateral creeping lesions typical of SC.

Patients usually consult because of loss of vision, metamorphopsias, and scotomata but less for photopsias. Vitritis can be associated with the choroiditis but anterior inflammation is usually not a feature. The active lesions appear as grey-yellow-white deep creeping lesions beginning in the posterior pole around the optic disc (Figure 23). The lesions are usually bilateral, but involvement is asymmetric. Curiously, the fovea seems to be spared for some time by the process, leading however to severe visual loss when it is ultimately involved.

**Figure 24:**
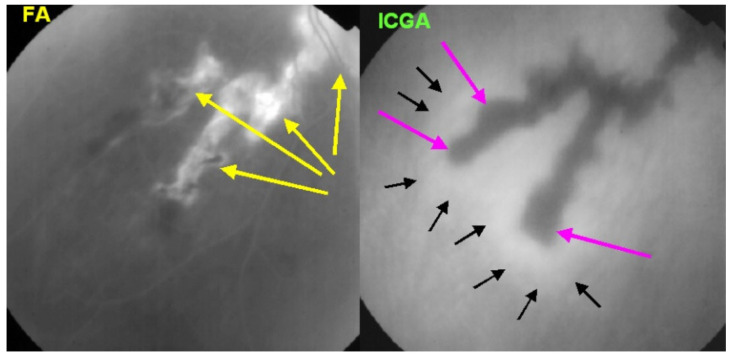
SC: comparison of FA and ICGA features in progressing disease. FA (**left**) shows hyperfluorescent areas resulting from window effect produced by atrophic chorioretinal areas (yellow arrows). ICGA (**right**) shows that atrophy ± active areas of disease is far more extended than supposed on FA (crimson arrows). Moreover, ICGA shows perilesional hyperfluorescence, indicating the progression of lesions (black arrows). Yellow arrows on FA show occult active areas revealed by ICGA.

Subretinal neovascular membranes occur in up to ¼th of cases. ICGA of old lesions shows mainly hypofluorescent areas up to the late angiographic phase, indicating chorioretinal scarring and atrophy [4]. In areas of active disease progression, ICGA shows hypofluorescent areas that go beyond the lesions seen on fundoscopy and/or fluorescein angiography (Figure 24). Another ICGA sign that can give information on disease activity is a diffuse perilesional hyperfluorescence [30] (Figure 24).

As for the other PICCPs, ICGA is more useful than FA to follow the evolution of lesions. FA of the active progressing edges appears as hypofluorescent in the early phase with progressive late staining, a pattern compatible with outer retina ischaemia. Older lesions appear as window defects associated with blockage caused by pigment clumping (masking effect), a typical pattern for chorioretinal atrophy and scars.

**Figure 25:**
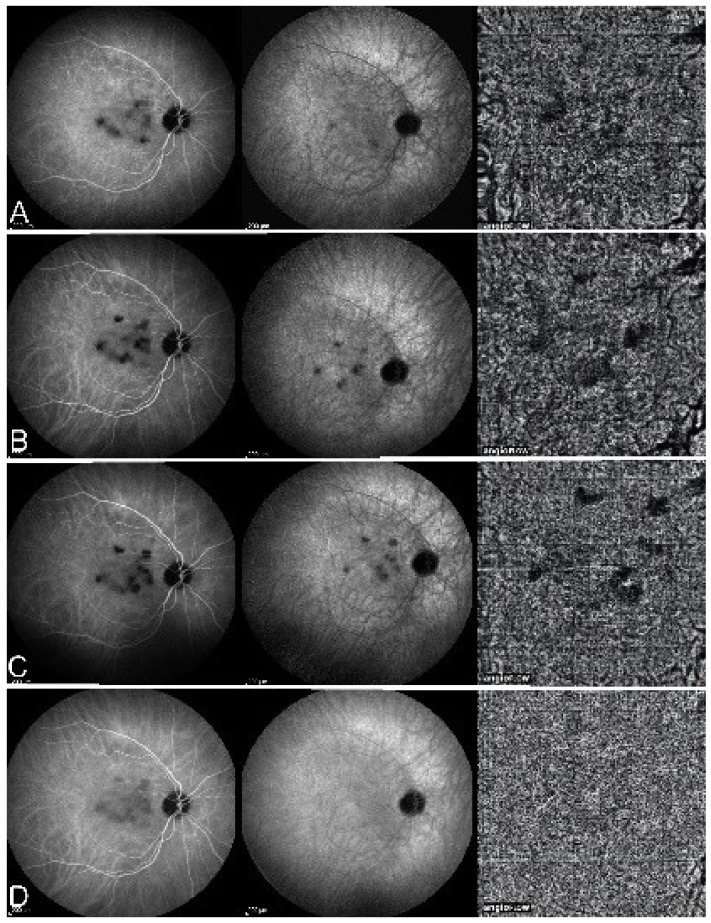
Serpiginous choroiditis—parallel images of intermediate phase ICGA (left column), late phase ICGA (middle column) and 6 × 6 mm OCT-Angiography (right column) of the right eye. (**A**). ICGA of the right eye reveals macular hypofluorescent lesions, increasing six months later (**B**), not responding to a Sub-Tenon’s injection (**C**), finally responding to the introduction of cyclosporine (**D**). (OCT-A pictures evolve in parallel with ICGA frames with faint dark areas visible at presentation ((**A**), right), more clearly visible 6 months later ((**B**), right) and three months after sub-Tenon’s injection ((**C**), right) and disappearing three months after cyclosporine treatment (**D**).

SD-OCT is useful to follow the borders of the lesions showing the condition of the different retinal layers often thickened by retinal oedema with hyperreflective accumulations in the outer retina with disruption of outer retinal layers. OCTA gives information on the vascular/choriocapillaris drop-out (Figure 25).

Functionally, VA depends on the location of the lesions, which is also the case for visual field testing. For lesions approaching the fovea, microperimetry is more useful than visual field testing.

Although treatment is entirely empirical, there is a growing body of evidence that dual and even triple steroidal and non-steroidal immunosuppression is probably the way to go [62,63]. However, before starting therapy, it is crucial to be sure that the patient has never been in contact with Mycobacterium Tuberculosis by performing an IGRA test (interferon-gamma release assay). The occurrence of CNVs has to be monitored and should be distinguished from progression of serpiginous lesions by ICGA and OCTA, if available.

In case of the development of neovascular membranes complicating serpiginous choroiditis, intravitreal anti-VEGF agents should be used additionally. An increase in corticosteroid and/or immunosuppressive therapy should also be considered [64].

##### Association of Different PICCPs in the Same Patient

Several articles have reported clinical cases combining two or more of the PICCP entities described here above. Association of MEWDS and multifocal choroiditis in the same patient has been described in several reports and evolution from MEWDS to MFC have been reported [65,66]. These and more reports represent a considerable body of evidence suggesting a common denominator for all the PICCPs, which has to be sought at the level of the choriocapillaris and pre-choriocapillaris vessels. Another link between these entities is represented by the common occurrence of blind spot enlargement due to peripapillary choriocapillaris non-perfusion in many of these cases [67,68,69].

##### Intermediary and Unclassifiable Forms of PICCPs

In addition to the cases that associate more than one clinical entity per patient, it is not rare to have intermediary forms that are difficult to classify within one or the other subset of PICCP. Either they have a hybrid presentation, or they have the morphology of one disease and the evolution of another, such as APMPPE with a disease course characterised with recurrences behaving like serpiginous choroiditis or there are atypical cases that cannot be classified within a determined subset but for which ICGA shows that the pathology is definitively situated at the level of the choriocapillaris.

**Figure 26:**
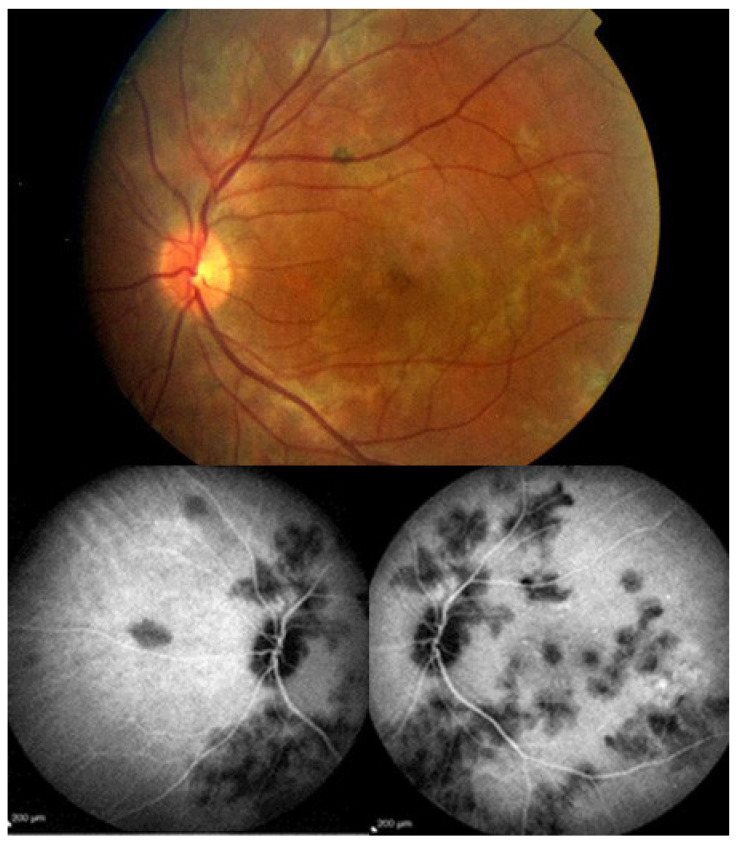
Case of Amppiginous Choroiditis. Disease started as APMPPE/AMIC lesions but progressed to become confluent resembling more serpiginous choroiditis. Extensive scarring was also more characteristic of SC.

When a sufficient number of cases of an intermediary or non-described form behaving similarly are gathered, this allows to describe a new entity which happened with a series of cases presenting as APMPPE having a subsequent evolution more compatible with serpiginous choroiditis first termed as Amppiginous choroiditis [70] (Figure 26) and later described as relentless placoid chorioretinitis [71]. In this report of six patients, the acute retinal lesions were similar to APMPPE or serpiginous choroiditis but had a prolonged progressive course and widespread distribution of lesions [71]. Along the same line of evidence, Gupta and colleagues, in one of the largest series of serpiginous choroiditis reported so far, indicate that in their part of the world, all cases having the initial features of APMPPE showed progression resembling serpiginous choroiditis during follow-up [61].

It is important to realise that PICCPs are a spectrum of diseases involving the choriocapillaris in diverse fashions and diverse degrees of severity and that even when the entity seems to be defined, it is important to perform a close follow-up. Some cases cannot be classified into one of the well-described entities but clearly present choriocapillaritis. For these cases, it is even more crucial to monitor them closely by SD-OCT and ICGA in order to intervene with inflammation suppressive therapy in case of worsening of the parameters.

**Figure 27:**
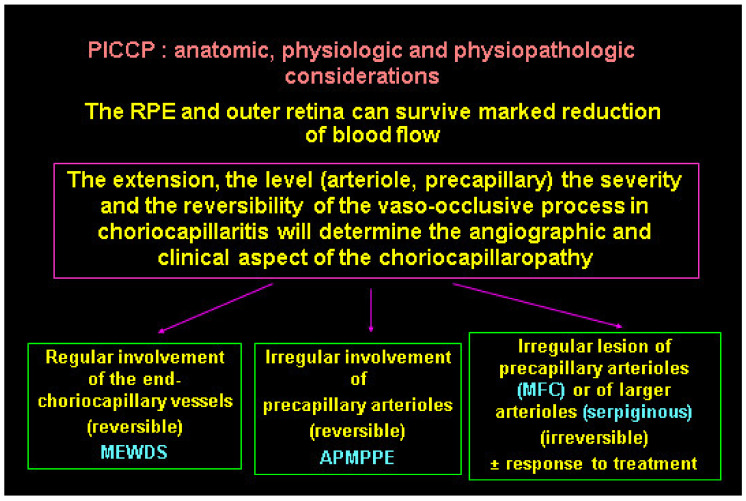
Clinicopathology hypothesis for the PICCPs. Type and extent of vascular involvement determine the clinical picture.

The diverse expressions of choriocapillaritis diseases could find an explanation in the particular haemodynamic properties of the choriocapillaris, a low flow circulating system which can easily close down. The severity of involvement probably also depends on the level, extension, and reversibility of vascular closure (Figure 27). If precapillary vessels are involved, lesions are more widespread with more ischaemic consequences, as seen in APMPPE/AMIC, MFC, and SC. In these cases, vascular drop-out is seen on OCTA. When capillary closure touches endcapillary vessels such as in MEWDS, ischaemic consequences are less pronounced and vascular drop-out cannot be seen on OCTA, as flow is extremely reduced, and its imaging is beyond the capacity of OCTA based on flow.

#### 3.1.2. Secondary Inflammatory Choriocapillaropathies

##### Acute Syphilitic Posterior Placoid Chorioretinitis (ASPPC)

**Figure 28:**
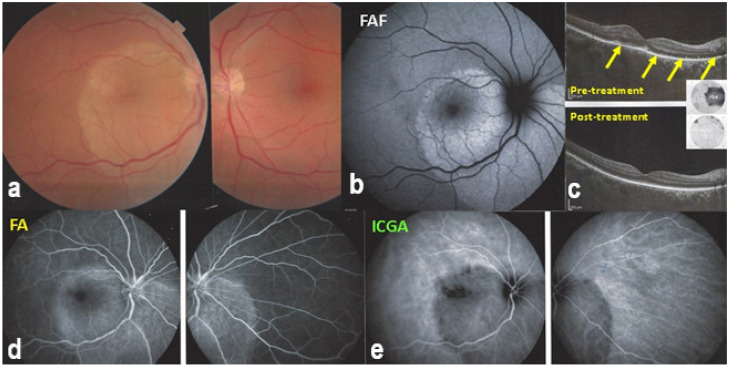
ASPPC (Acute Syphilitic Posterior Placoid Choroiditis) Posterior placoid lesion (**a**), hyperautofluorescent (FAF, (**b**)) due to loss of photoreceptor outer segments (OCT—(**c**)) and better visualization lipofuscin in RPE. On FA, the placoid lesion is hyperfluorescent due to ischaemia of outer retina because of choriocapillaris non-perfusion appearing hypofluorescent on ICGA (**d**,**e**). SD-OCT (**c**) shows loss of photoreceptor outer segments (yellow arrows) corresponding to visual field loss (insert **c-top**). On the lower post-treatment SD-OCT, outer segments reconstitute with improvement of visual field (insert, **c-bottom**).

In 1990, Gass (once more) described a particular form of syphilitic posterior involvement which he called acute syphilitic posterior placoid chorioretinitis [72]. This clinical picture resembled APMPPE, which he had described 22 years earlier, which is not astonishing, as we know now that the mechanism is choriocapillaritis [22,73,74,75]. As the trigger (Treponema Pallidum) causing choriocapillaritis is known for this entity, it is classified within the secondary inflammatory choriocapillaropathies [22]. Indeed, the lesions are not caused directly by the infectious agent but through an immune process inducing choriocapillaritis [23,76] (Figure 28).

##### Tuberculosis Related Serpiginous Choroiditis (Serpiginous-like Choroiditis, Cerpiginoid Choroiditis, Multifocal Serpiginous Choroiditis)

Our colleagues in India first described serpiginous choroiditis associated with exposure to Mycobacterium Tuberculosis. Patients were of the same young adult age group as all other PICCPs [60,61].

They called these cases serpiginous-like choroiditis or serpiginoid choroiditis. In this case also, choriocapillaritis is triggered but not directly caused by the infectious agent. These cases of choriocapillaritis are considered to be immune-mediated. Therefore, therapy should combine multiple anti-tuberculous agents in association with immunosuppressive treatment.

#### 3.1.3. Conditions Often Associated with Choriocapillaritis but Where Choriocapillaris Is Not Involved or Has No Primary Role

##### AMN (Acute Macular Neuroretinitis)

**Figure 29:**
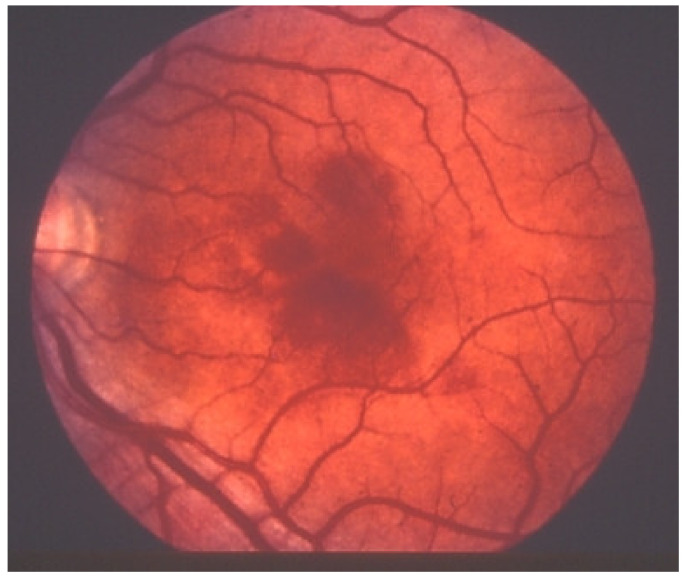
AMN. Fundus appearance. Petaloid lesion.

Acute macular neuroretinopathy (AMN) is a rare, outer retinal disease of unknown origin affecting typically young women who complain of paracentral scotomas mono or bilaterally and often occurring with oral contraceptive use. Visual loss is variable. As described by Bos and Deutman [77], the fundus examination reveals distinctive lesions characterized by macular reddish-brown, wedge-shaped lesions corresponding to scotomas referred by the patients (Figure 29).

**Figure 30:**
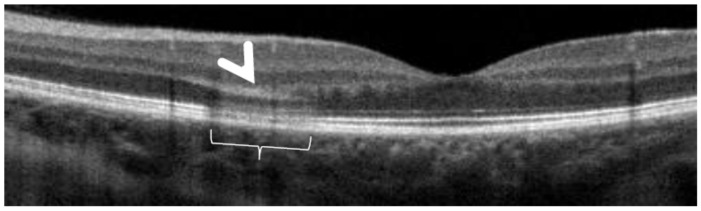
AMN. SD-OCT image performed one day after the onset of symptoms. SD-OCT shows a highly reflective band at the level of the OPL/ONL (arrowhead), resembling Paracentral Acute Middle Maculopathy (PAMM) with alterations of EZ and IZ lines (bracket).

**Figure 31:**
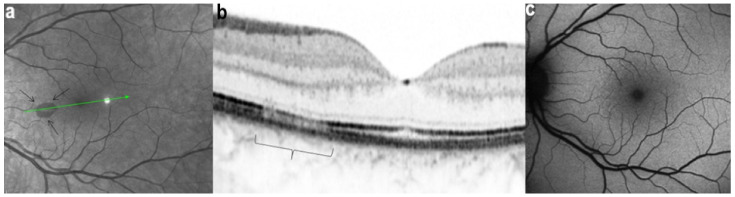
AMN. NIR-R, SD-OCT and FAF images at eight days after the onset of symptoms. (**a**): NIR-R imaging shows a sharply oval hyporeflective area (arrows) corresponding, on SD-OCT (**b**), to an attenuation of the EZ whereas the IZ is not seen (b, bracket). (**c**): BAF signal is normal.

**Figure 32:**
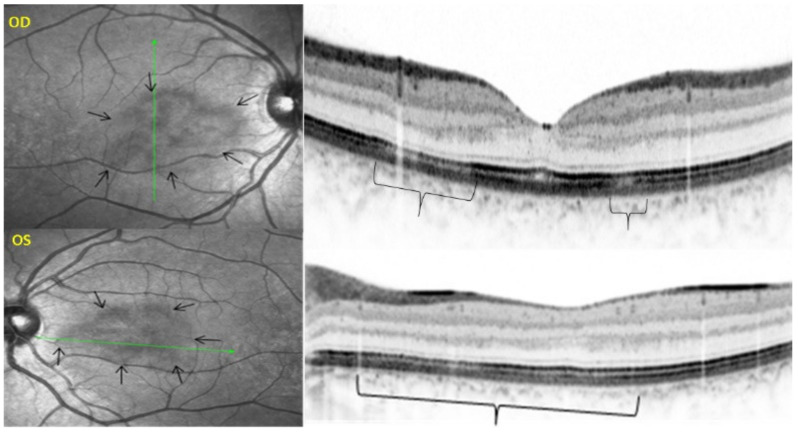
AMN. NIR-R, SD-OCT, FAF, FA, and ICGA images at first presentation. NIR-R and SD-OCT images: NIR-R imaging shows hyporeflective petal-like oval areas in the macular region (arrows) in corresponding on SD-OCT to a reduction of the signal of the IZ (brackets). In OD, the reduction of the signal is limited to two segments of the outer retina, while in OS, it comprises the whole outer retinal section corresponding to the hyporeflective infrared lesion.

However, these typical lesions are not always clearly visible on fundoscopy while they are seen more precisely when using the near infrared reflectance (NIR-R) imaging mode that demonstrates sharply demarcated, hyporeflective lesions more definite than those found on fundoscopy [78]. Blue-light fundus autofluorescence (488 nm, lipofuscin related) (BAF), fluorescein angiography (FA) and indocyanine green angiography (ICGA) are usually not significant, while near infrared autofluorescence (787 nm, melanin related) (NIR-FAF) can show abnormalities [78]. Together with the NIR-R, the spectral-domain optical coherence tomography (SD-OCT) represents a very useful new imaging technology to diagnose AMN demonstrating that the outer retina is primarily affected. In the early phase of the disease, OCT shows a transient, localized hyperreflectivity at the outer plexiform layer (OPL)/outer nuclear layer (ONL) [79] (Figure 30). In a short time, the hyperreflectivity is replaced by focal abnormalities of the ellipsoid zone (EZ) and interdigitation zone (IZ) that are often associated with thinning of the ONL (Figure 31 and Figure 32) In addition, electrophysiologic analysis also with multifocal electroretinography has clearly localized the lesion impact at the level of the outer retina [80]. AMN has good prognosis with a slow, progressive improvement of the symptomatology. Macular lesions can persist for a long time.

AMN is often put in the group of choriocapillaritis diseases, which is not the case. It is a rare macular disease of the outer retina. It can be unilateral or bilateral with an accompanying paracentral or central scotoma with or without visual loss. BAF, FA, and ICGA are mostly normal. Retinal capillary ischaemia has been proposed as a possible mechanism. SD-OCT findings suggest that the outer retina is the location of the pathology including hyper-reflectivity of the OPL and ONL, disruption of ellipsoid zone (EZ), and interdigitation zone (IZ) and subsequent thinning of the ONL. The introduction of OCTA has more clearly demonstrated retinal aetiology. As the patients are mostly young women, the main differential diagnosis for AMN is retrobulbar optic neuritis.

##### UAIM (Unilateral Acute Idiopathic Maculopathy)

**Figure 33:**
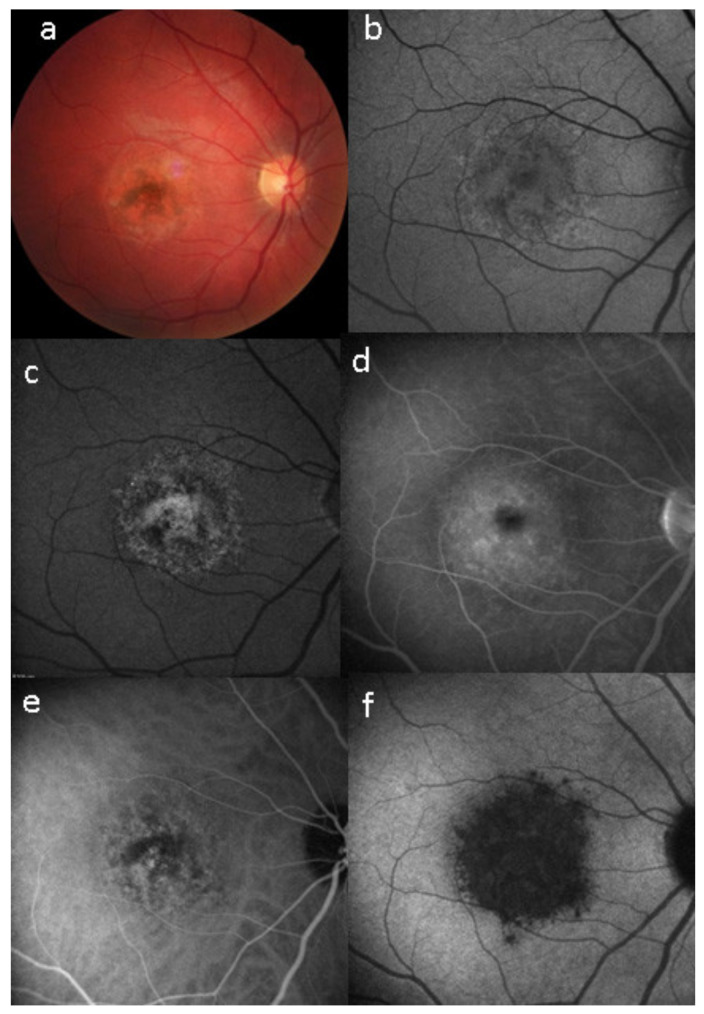
Case of UIAM at presentation: (**a**) fundus photography showing a reddish foveal detachment surrounded with an irregular, circular area of greyish RPE discoloration. (**b**) BL-FAF shows a light hypoautofluorescence in the macular area. (**c**,**d**) FA early and late phases reveal late hyperfluorescence due to the leakage and pooling in the neurosensory foveal detachment. (**e**,**f**) ICGA early and late phases reveal persistent hypofluorescent area surrounded, in the late frame, by a hyperfluorescent ring, sign of hyperpermeability of the choroidal vessels.

**Figure 34:**
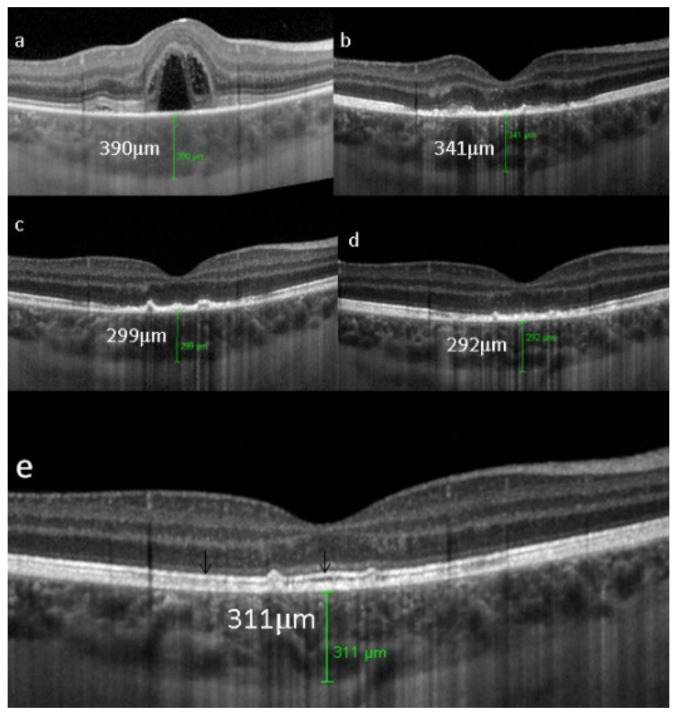
UAIM. EDI-OCT findings from one day after symptom onset to one year later. (**a**) at presentation, a neurosensory dome-shaped foveal (bacillary layer) detachment with compartmentalized subretinal turbid fluid and a choroidal thickening beneath the fovea. (**b**) a week later, complete resolution of the foveal detachment, damage of the outer retina with the loss of the ellipsoid zone and reduction of the choroidal thickness. (**c**) three weeks later, the outer retinal boundary is less irregular and the choroidal thickness is reduced. (**d**) two months later, further reorganization of outer retinal structures without the visualization of the ellipsoid zone. (**e**) one year later, there is an almost complete normalization of the outer retina with the recovery of the ellipsoid zone and interdigitation zone (black arrows). The choroidal thickness (green cursor) remains low compared to onset of the disease.

Unilateral Acute Idiopathic Maculopathy (UAIM) is a rare, macular disease first described by Yannuzzi [81]. It affects young people presenting with an acute, serious, unilateral central vision loss. The aetiology of UIAM is still unclear but the involvement of Coxsackievirus infection has been documented by multiple reports connecting UIAM with Hand-Foot-Mouth Disease [82]. The fundus examination reveals a distinctive clinical picture, including irregular greyish discoloration of the macular area characterized, at its first appearance, by an exudative retinal detachment that can resolve spontaneously in a few days or, otherwise, within one to two months of the onset, leaving a residual “bulls-eye”-like macular appearance [83,84] with a good recovery of the visual acuity. In some patients, papillitis, subretinal exudates, intraretinal haemorrhages, and vitreous flare have also been noted [81]. Fundus autofluorescence shows, once the exudative macular detachment has regressed, a bright autofluorescence, sign of an increase of fluorophores due to the amount of cellular debris and reactive hypertrophy/hyperplasia of the RPE [85,86] (Figure 33). Later in the disease course, this mottled hyperautofluorescence progressively shifts from hyperautofluorescence to hypoautofluorescence, suggesting RPE loss. Fluorescein angiography shows increased hyperfluorescence with subsequent pooling in the macular area. In the convalescent phase of the disease, FA shows resolution of the subretinal leakage with persistence of early, mottled hyperfluorescence due to “window-effect” caused by the damage to the RPE in addition to (late) staining. ICGA shows a hypofluorescent dark area, more evident in the late frames, often surrounded by a halo of late, increased hyperfluorescence, sign of choroidal hyperpermeability [87,88]. During the follow up, the ICGA hypofluorescent macular area becomes progressively less dark, indicating an improvement of the choriocapillaris perfusion. SD-OCT shows neurosensory macular detachment with hyperreflective subretinal material and EDI-OCT [83] reveals a sub-foveal increase of the choroidal thickness. With the regression of the macular detachment, SD-OCT shows irregularity and disruption at the level of the photoreceptors and RPE layers and EDI-OCT shows a progressive reduction in choroidal thickness [89]. A gradual recovery of the outer retina integrity occurs during the remission phase of the disease (Figure 34).

UIAM has a good prognosis with spontaneous recovery of the visual acuity and OCT findings, although a mottling of the RPE beneath the fovea persists. Choriocapillaris involvement seems to be secondary to a choroidal inflammation/vasculitis sufficiently severe to involve the RPE.

**Figure 35:**
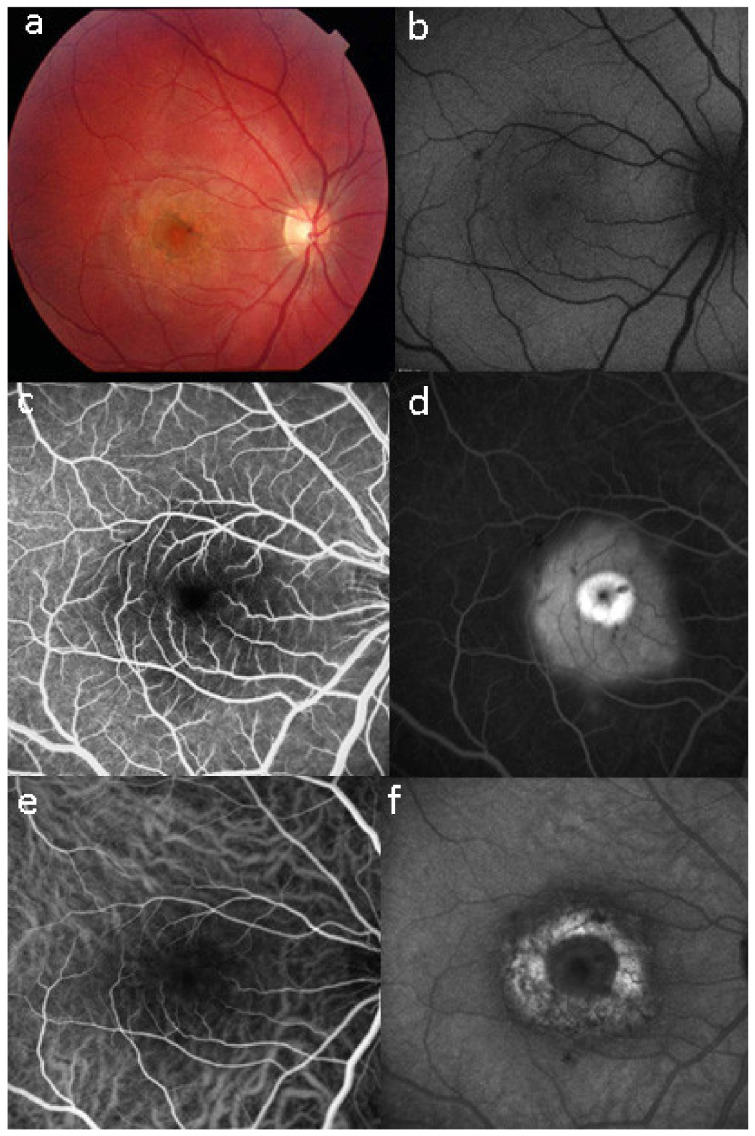
Images of a case of UIAM a week after the onset of symptoms. (**a**) fundus photography showing well-circumscribed area of RPE atrophy and hyperplasia. (**b**,**c**) BAF and NIR-FAF show stippled hyperautofluorescence, more evident in NIR mode, indicating increase not only of lipofuscin but especially of melanin and compounds closely related to melanin. (**d**) FA in the late phase shows hyperfluorescence (staining). (**e**,**f**) ICGA early and late phases reveal only in the late phase of the angiography a large area of hypofluorescent dark area, sign of choriocapillaris ischaemic non-perfusion.

Multimodal imaging shows that UAIM is not a primary choriocapillaritis but the choriocapillaris is only involved secondarily. It also shows that the RPE is damaged, which is not the case in the PICCPs, except in severe cases. Following is the sequence of events in UIAM by multimodal imaging: ICGA shows initial inflammatory hyperpermeability of larger choroidal vessels, leading to increased choroidal thickness seen on EDI-OCT and choriocapillaris non-perfusion (late phase ICGA hypofluorescence) causing major outer retinal and RPE disturbance that slowly recovers over time. It is therefore thought that UIAM is an acute choroidal vasculitis causing secondary choriocapillaris ischemia, in turn damaging the RPE and outer retina (Figure 35).

##### AZOOR (Acute Zonal Occult Outer Retinopathy)

Acute zonal occult outer retinopathy (AZOOR) was first described by JD Gass in 1992 [90] and a more extensive article was published in 2002, including 51 cases [91]. He described a disease characterised by the sudden onset of subjective scotomas and photopsias due to loss of areas of outer retina with a normal fundus aspect.

**Figure 36:**
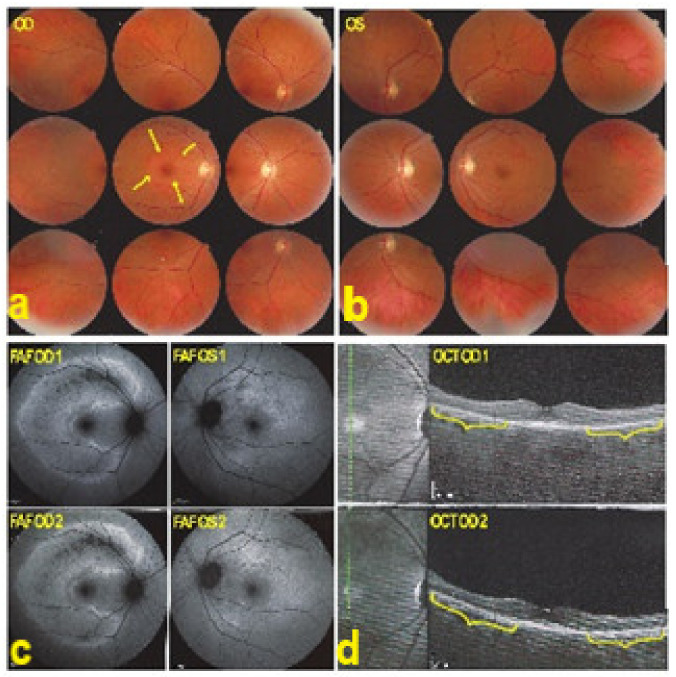
AZOOR. Fundus photograph showing a pale ring around the fovea OD (arrows) (**a**) and normal OS (**b**). In (**c**) Fundus autofluorescence (FAF) shows an extensive C-shaped hyperautofluorescent area around the fovea with dots of hypoautofluoresce within this zone OD. (FAF OD 1) Left hyperautofluorescence parapapillary inferiorly and supero-temporally to the fovea. (FAF OS 1), corresponding to the loss of photoreceptors on SD OCT, extensive in OD (brackets in OCT OD 1 (**d**)), with a stable evolution after infliximab therapy (FAFOD/OS 2 and brackets in OCT OD 2 (**d**)).

**Figure 37:**
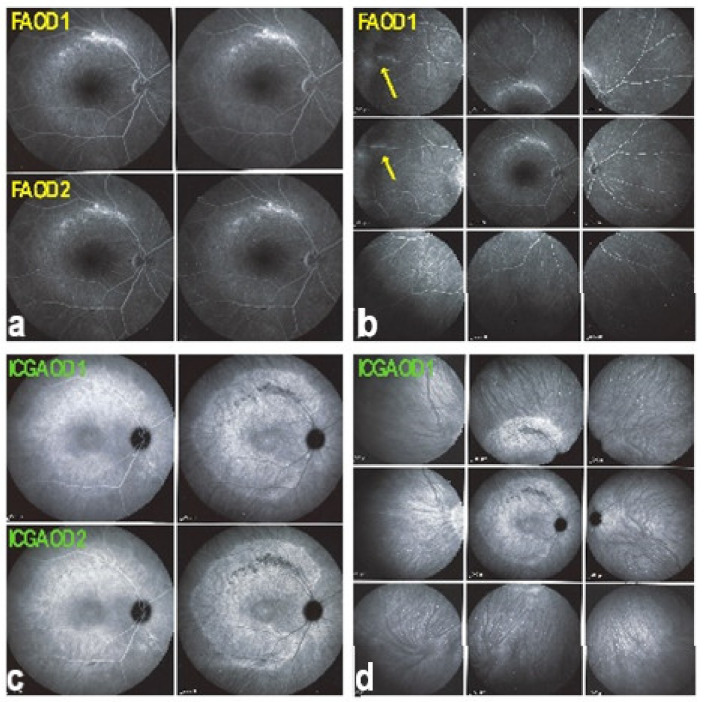
AZOOR. FA faint hyperfluorescence corresponding to the C-shaped hyperautofluorescent area on FAF OD ((**a**), FA OD 1), including a zone inside this area of brighter hyperfluorescence (window defect), indicating chorioretinal atrophy. (a, FA OD 1). In the periphery on the right panorama supero-temporal vasculitis ((**b**), FA OD 1, (arrows)). Late ICGA frames show hyperfluorescence corresponding to the C-shaped hyperautofluorescent areas on FAF, indicating preservation of choriocapillaris, except in the area along the superior temporal arcade, which was hypofluorescent corresponding to hypoautofluorescence on FAF and hyperfluorescence on FA, all images indicating chorioretinal atrophy (ICGA OD 1, (**c**), and ICGA OD 1, (**d**)). Images remained stable after infliximab therapy (a FA OD 2 and c, ICGA OD 2).

AZOOR affects young- to middle-aged patients, is largely predominant in women, and starts with an acute onset of visual field defect in one or both eyes associated with photopsias, decrease of contrast sensitivity and photophobia. The fundus is essentially normal at presentation, but later can present pigment clumping with progression of retino-choroidal atrophy. Visual field defects remain at best stable when the disease stabilises within 4–6 months but can also progress in a proportion of patients. The aetiology is speculative, including the hypothesis of the involvement of an infective viral agent with subsequent autoimmune alteration of the photoreceptors. Treatment is empiric, including systemic steroids and/or immunosuppressive agents [92]. Gass speculated that AZOOR was part of a spectrum of diseases, including MEWDS, AIBSE, MFC, and other sub-entities that used to be grouped under the term of “White Dot Syndromes” and now known to be diseases of the choriocapillaris [90,91,93]. This assumption by Gass was put forward as the patient characteristics of these inflammatory choriocapillaropathies were similar to those of AZOOR patients and because there was a number of reports in which choriocapillaritis patients were subsequently found to present AZOOR [92]. Reports on such associations continue to be published, indicating that patients can be exposed to both types of disease, which does not necessarily imply that they are caused by the same mechanisms [94,95,96]. With multimodal imaging, it became clear that the disease is a retinopathy and choriocapillaris is not involved, at least not primarily. As indicated by Gass, fundus examination is usually normal. Diseased areas are hyperautofluorescent on BAF, which correspond to loss of photoreceptor outer segments on SD-OCT (Figure 36). On FA, diseased areas are discreetly hyperfluorescent (except areas of chorioretinal atrophy which are hyperfluorescent due to window-effect), while on ICGA, these areas are slightly hyperfluorescent indicating choriocapillaris integrity (except areas of chorioretinal atrophy that are hypofluorescent) (Figure 37).

### 3.2. Stromal Choroiditis Entities

**Figure 38:**
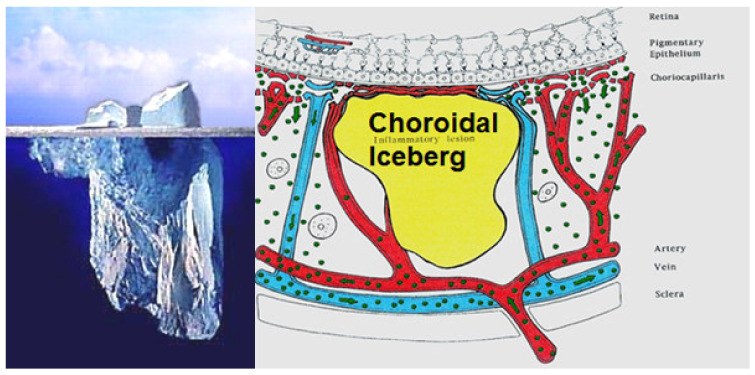
Cartoon of choroidal lesion and the analogic concept of iceberg effect.

The main lesion process at the base of stromal choroiditis entities is the choroidal focus (granuloma), which impairs diffusion of the ICG dye and is seen in a negative, dark, hypofluorescent fashion and can be assimilated to an iceberg as most of its mass is occult, except to ICGA (Figure 38).

The relevant angiographic times are the intermediate (±8–10 min and late (±20–25 min) phases, during which the physiological extrusion occurs and sheds into the choroidal stroma (Figure 2). Additional ICGA angiographic signs are listed on Table 1.

Stromal choroiditis entities are further sub-classified into primary stromal choroiditis where the origin of inflammation is obligatorily in the choroidal stroma, such as for BRC and VKH, as opposed to secondary stromal choroiditis where the choroidal stroma is only the chance innocent bystander of a systemic disease, such as sarcoidosis. In primary stromal choroiditis, the choroid is relatively homogenously involved whereas in secondary the involvement is random, as seen in sarcoidosis.

#### 3.2.1. Primary Stromal Choroiditis

##### HLA-A29 Birdshot Retinochoroiditis (BRC)

Birdshot retinochoroiditis is a rare, bilateral, retinochoroidal, inflammatory disease without known systemic involvement; it is mostly seen in Caucasians [97]. It was first described in 1980 by Ryan and Maumenee [98]. In 1981, Gass described 11 similar cases and called the disease vitiliginous chorioretinitis [99]. This posterior uveitis with dual, independent retinal and choroidal inflammation has no known extraocular inflammation sites and yet features the strongest-known HLA association, HLA-A29, present in close to 100% of cases. Because of this very strong association with crucial diagnostic value, the disease should better be called HLA-A29 BRC. It affects middle-aged patients with a slight predominance of women. Anterior chamber inflammation is scarce at most but vitritis is often moderate to substantial with formation of strands. The particularity of the disease is that retinitis and vitritis develop independently from choroidal inflammation, the latter being a typical stromal choroiditis. During long years, the appraisal of BRC was hampered by inadequate diagnostic criteria, which were rectified in 2018 (Table 5) [100,101].

**Figure 39:**
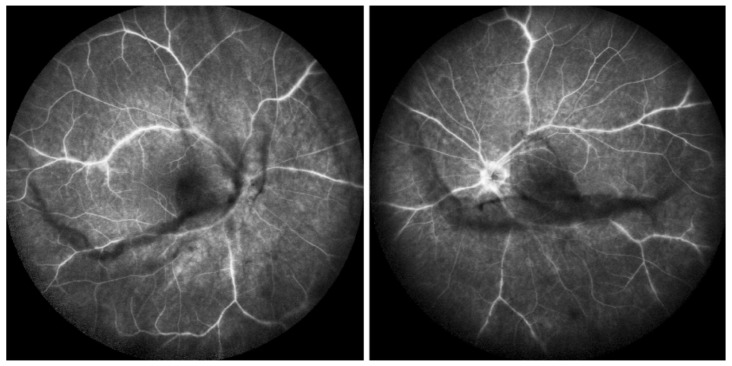
Retinal and vitreous involvement in BRC. Large strands of vitreous opacities and widespread retinal vasculitis.

Retinal signs observed on fundus examination can include retinal vasculitis of large veins and papillitis, but retinal disease is most clearly demonstrated by FA. Patients usually present with vitreous symptoms that include floaters and fuzzy vision, as well as retinal symptoms such as dimness of vision, fluctuating vision, subjective scotomas, and peripheral vision difficulties. In the presence of vitritis, these symptoms lead the clinician to perform FA and OCT, which best illustrate retinal involvement (Figure 39).

**Figure 40:**
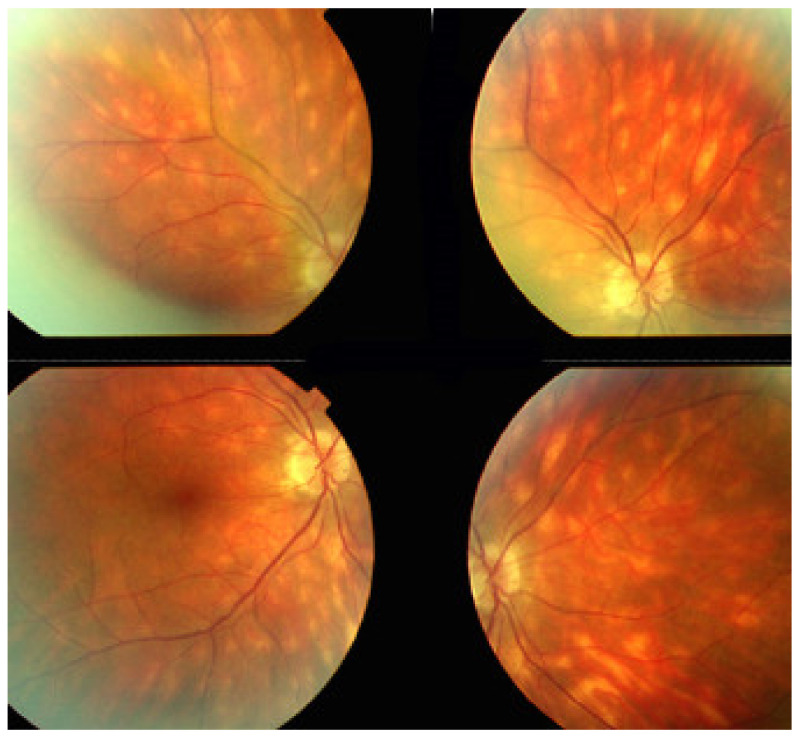
Choroidal involvement in BRC. Typical rice-shaped discolored areas.

Rice-shaped depigmented BRC fundus lesions were the hallmark sign that allowed the identification and discovery of this entity and turned out to be one of the main disease-defining criteria (Figure 40).

**Figure 41:**
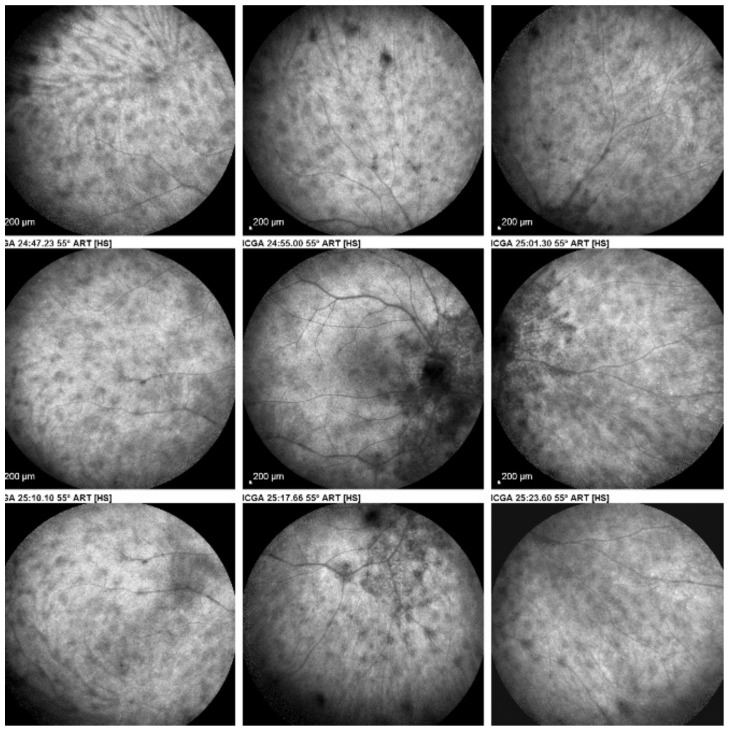
ICGA in BRC. Even round HDDs regularly distributed over all the fundus.

It was not possible to explore choroidal involvement in more detail at the time when the disease was initially described, as ICGA became available only in the early 1990s. Today, after the advent of ICGA and the histopathological findings reported by Gaudio et al. [102], it is clear that choroidal inflammation in BRC belongs to the category of primary stromal choroiditis, which also includes VKH disease and sympathetic ophthalmia. Unlike these two latter conditions (which are purely primary stromal choroiditis), BRC is indeed a primary stromal choroiditis but not only, as additional unrelated retinitis is also present. Choroidal ICGA lesions appear very early in the disease but remain occult unless ICGA is performed or until BRC fundus lesions appear. Choroidal involvement is best demonstrated by ICGA [103,104] (Figure 41).

BRC should undoubtedly be classified as a granulomatous uveitis. This assessment is based clinically on the granulomatous KPs observed in 15% to 20% of cases and the mutton-fat precipitates on the detached posterior vitreous described by Gass [105]. The histopathology report by Gaudio et al. also described granulomatous features in the infiltrates around the islets of stromal melanocytes [102].

**Figure 42:**
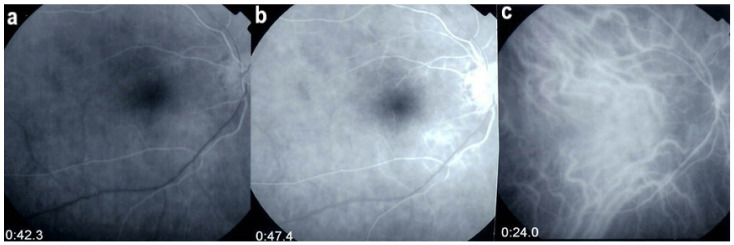
Pseudo-delay in retinal circulation. A patient in the exudative stage of BRC exhibits no fluorescein marking of large veins at 42.3 s. (**a**) and 47.4 s (**b**) after injection. This is not due to an increase of arterio-venous circulation time, but to massive extrusion of the small fluorescein molecule from retinal capillaries and larger vessels. This phenomenon is revealed by ICGA; opacification of large veins occurs normally at 24.0 s (**c**), as the large ICG complex does not extrude from retinal vessels.

Multimodal imaging allowed precise appraisal of BRC and allowed to understand the disease mechanisms. Fundus photography is marginally useful to follow BRC evolution as they indicate stromal scars while ICGA is the most sensitive modality to document disease activity (Figure 41). When treated early, based on ICGA findings, fundus birdshot lesions may never appear. As far as FA is concerned, during the early exudative stage of BRC, three characteristic signs are observable using FA. In the early angiographic frames, there is “an increased retinal circulation delay”, which is in fact a pseudo-delay explained by the diffuse capillary exudation of fluorescein to the extent that there is not enough dye to normally mark the large veins. In contrast, the large ICG complex does not leak from retinal capillaries and normally marks large retinal veins, which indicates that there is no real hemodynamic slowing (Figure 42). Consequently, the first specific FA sign in BRC is massive and diffuse retinal capillary leakage with diffuse retinal edema, which was already observed by Gass [106] (Figure 42).

**Figure 43:**
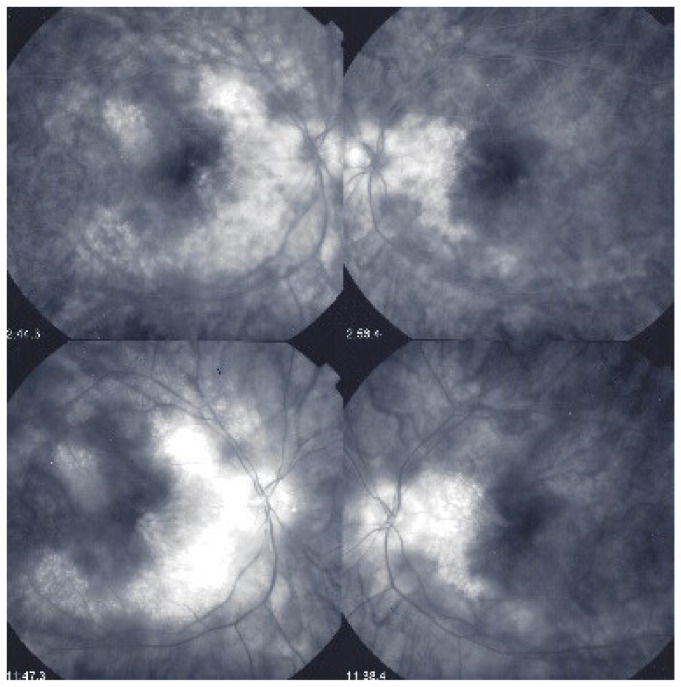
Foveal sparing of diffuse retinal edema. This patient was followed for more than seven years for “neuroretinitis”, without treatment. There is massive retinal oedema that also involves the posterior pole, yet the fovea remains relatively spared, which explains the patient’s preserved visual acuity, while visual fields were severely impaired (Figure 44).

**Figure 44:**
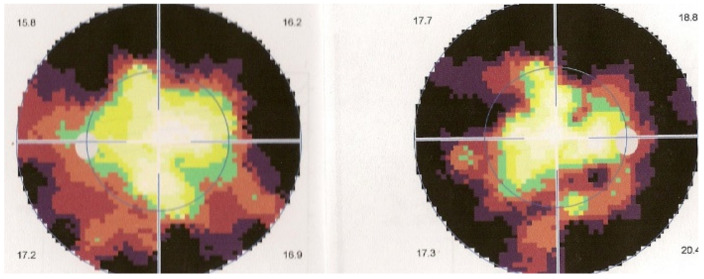
Visual field impairment due to diffuse retinal oedema of posterior pole while fovea is spared. Explains why BRC patients retain a full visual acuity while visual fields are severely impaired.

This diffuse retinal edema also involves the macula, but strangely spares the fovea, which explains the good vision retained by a large proportion of patients for a relatively long period of time (Figure 43 and Figure 44). On later frames, there is leakage along large veins, representing the second significant FA sign in BRC (Figure 39). The third sign is disc hyperfluorescence, which is almost always present (Figure 43).

**Figure 45:**
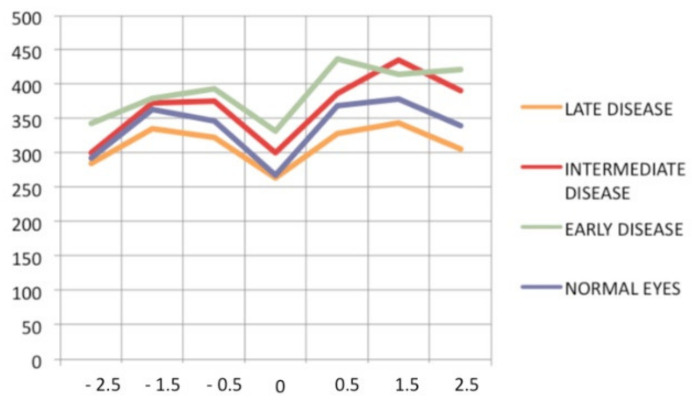
Progression of retinal disease as followed by optical coherence tomography (x axis indicates distance from fovea (=0); y axis indicates thickness of retina in microns). Early disease (green line) features thickening of the retina (and to a lesser degree, the fovea). In late disease (yellow line), there is diffuse thinning, except in the fovea; foveal thickness remains comparable to normal (purple line) (from Ophthalmic Surg Lasers Imaging 2012; 43-suppl S25–31).

Retinal SD-OCT allows another approach and provides a morphological explanation for the retinal signs observed using FA. We showed that in early disease, a diffusely thickened retina was measured in the macula, but less so in the fovea, which exhibited fewer fluctuations in thickness during disease progression. This supports the FA finding showing that the fovea was relatively spared [107], while in late disease, the mean retinal thickness was reduced (mostly in patients for whom treatment had been delayed) (Figure 45).

Optical coherence tomography also provided information about the vitreo-retinal interface; it revealed the presence of thin, pauci-contractile epiretinal membranes in up to 93% of cases [107].

EDI-OCT is an essential imaging modality to follow choroiditis by measuring thickness which is substantially increased in acute disease but decreases over time [108]. This imaging modality is however less reliable than ICGA, as during evolution, active areas producing thickening can coexist with atrophic evolution and activity is best identified by ICGA.

Relevant ICGA signs for diagnosis and disease monitoring are the presence of HDDs and fuzziness of large choroidal vessels (Figure 41) HDDs were suspected to be caused by stromal inflammatory foci and this hypothesis was confirmed histopathologically by Gaudio et al. [102]. Fuzziness of choroidal vessels indicates vasculitis of large choroidal vessels. ICGA signs are present before the appearance of characteristic BRC fundus lesions and ICGA is therefore essential for early diagnosis [103]. In case of early diagnosis thanks to ICGA and early treatment, the rice-shaped birdshot lesions will never develop [109].

OCTA can show interesting images but BRC being a stromal disease, it will be of little practical use. The most useful functional test is not visual acuity but visual field testing. In a recent report, we showed that all patients diagnosed with BRC presented visual field disturbance, without exception [97]. Beside imaging, in case of suspected BRC, it is essential to perform HLA-testing, which, in such a situation is diagnostic if present.

**Figure 46:**
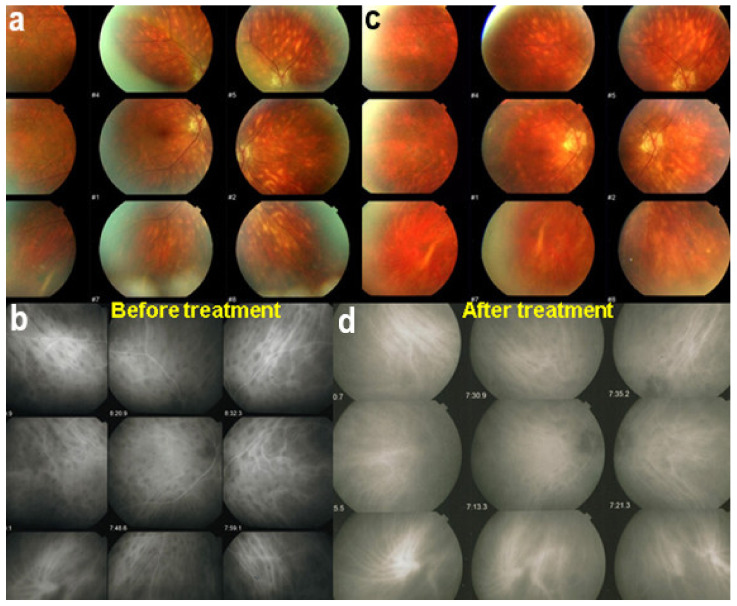
ICGA lesions. Fundus examination is best to monitor disease activity (right eye). This patient accepted treatment with delay with numerous hypopigmented birdshot lesions. After applying immunosuppressive therapy, the HDDs, numerous before treatment (**b**) responded to treatment (**d**), while no fundus change was seen (**a**,**c**), as birdshot fundus lesions correspond to stromal scars.

There is increasing evidence that aggressive and sustained treatment is probably needed in the majority of patients [110]; no more than 10% to 15% of patients present a relatively mild course [111]. Once the diagnosis is established, the criterion to treat is the presence of visual field defects. If involvement is unilateral, then the introduction of systemic immunosuppressive treatment can sometimes be deferred for a period of time by using sub-Tenon’s injections of triamcinolone acetonide (40 mg per injection) every 4–6 months if the other eye is not functionally affected. Once systemic treatment is decided, a combination of systemic steroids with immunosuppressants, mostly mycophenolic acid (Myfortic^®^), is strongly recommended. In case of insufficient recovery of visual fields and/or persistence of retinal inflammatory signs (mainly monitored by FA), a second immunosuppressant or biologic agent is added (for example, anti-TNF-α agents). Early treatment is usually the rule. However, at present, treatment is too often delayed because of a delayed diagnosis or because patients decline treatment. We showed that insufficient or delayed treatment results in retinal and choroidal atrophy, as evidenced by OCT and EDI-OCT. In patients who are treated before the development of BRC fundus lesions, the appearance of such lesions can mostly be prevented (Figure 46).

BRC is a granulomatous ocular inflammatory disease that seems to be limited to the eye with a close to 100% association with the leukocyte histocompatibility antigen HLA-A29. Dual parallel non-related inflammation of the retina and the choroid is particular to BRC. Retinal involvement is responsible for substantial clinical morbidity if the disease is left untreated. Treatment must be started early and should be vigorous and prolonged. Choroidal involvement responds rather well and rapidly to steroidal and non-steroidal immunosuppression; the development of depigmented BRC fundus lesions can even be prevented if treatment is started early. The impact of therapy on retinal disease is more limited but can likely avoid retinal atrophy if treatment is applied early. However, ICGA is crucial to achieve early diagnosis before the appearance of what has thus far been considered the hallmark of BRC: rice-shaped choroidal depigmented fundus lesions.

###### Vogt-Koyanagi-Harada Disease (VKH)

Vogt-Koyanagi-Harada (VKH) disease is a bilateral, granulomatous panuveitis with exudative retinal detachments associated with systemic manifestations, such as meningeal signs and cutaneous signs (poliosis, alopecia, vitiligo) and dysacusis [112]. There is now enough evidence indicating that the disease is caused by an autoimmune process against melanocytes or antigens present in these cells [113]. The disease is more prevalent in Asians, in particular Japanese; in Hispanics; and native Americans, but can occur in any individual of any race.

Histopathologic evaluation reveals thickening of the choroid with stromal cellular inflammation constituted by macrophages, lymphocytes, and epithelioid cells containing melanin. Chronic inflammation and recurrences lead to the loss of choroidal melanocytes and to the appearance of sunset glow fundus [114].

The primary insult is known to occur at the level of the stromal melanocytes of the choroid. The lesion process in this disease defines VKH as a strictly primary choroidal stromal inflammatory disease and involvement of the adjacent structures is only secondary to choroidal disease. VKH is therefore the typical example of a primary stromal choroiditis as is the case for sympathetic ophthalmia and HLA-A-29 birdshot retinochoroiditis.

**Figure 47:**
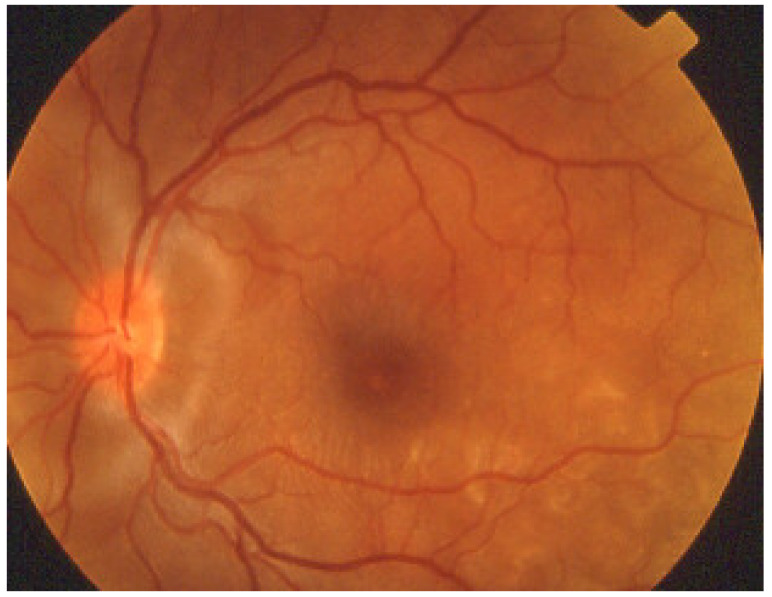
VKH exudative retinal detachments. Fundus photograph showing posterior peripapillary and parafoveal temporal inferior exudative retinal detachments.

**Figure 48:**
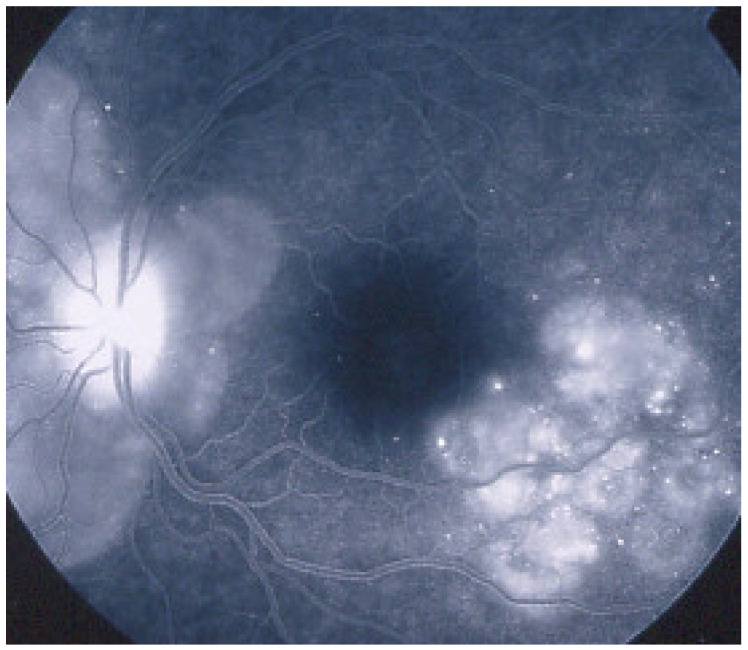
VKH exudative retinal detachments. FA showing posterior peripapillary and parafoveolar temporal inferior exudative retinal detachments.

VKH disease is newly separated into two phases [1,115]. Initial-onset disease starts with a prodromal stage, including flu-like symptoms, headache, tinnitus, and vertigo, followed within a few days by an acute ocular initial-onset phase, consisting of an acute uveitis with optic disc hyperaemia and swelling, and characteristic multifocal exudative serous retinal detachment (Figure 47 and Figure 48).

Lesions are bilateral but can be asymmetric, the second eye being sometimes involved with delay [116]. So-called unilateral involvement are always cases where appropriate choroidal investigations for occult disease have not been performed [2,116]. If appropriate immunosuppressive treatment is not introduced at this initial-onset phase, disease will evolve in many cases into the chronic phase of disease, difficult to treat and needing prolonged management with many complications, including, among others, glaucoma and cataract. With chronic evolution, diffuse retinal pigment epithelium alterations classically appear as “salt-and-pepper” fundus appearance and sunset glow fundus [117].

**Figure 49:**
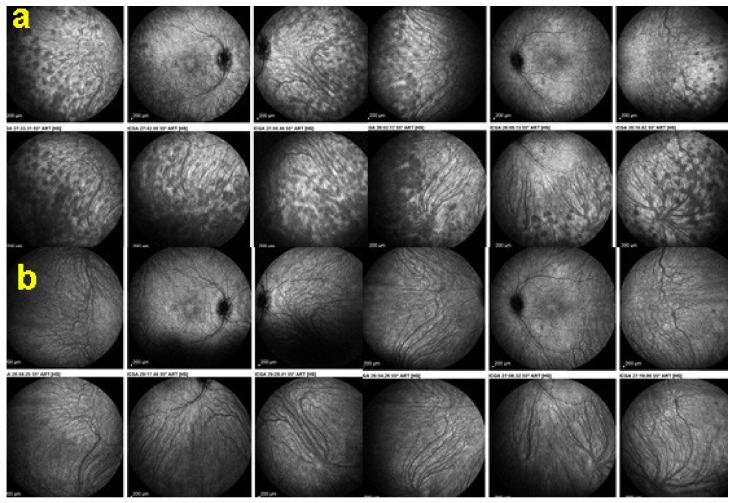
ICGA in VKH disease. Regular distribution all over the fundus of evenly sized HDDs before treatment (**a**) having mostly disappeared after the introduction of dual steroidal and non-steroidal immunosuppression (**b**).

Among the multimodal imaging approach, ICGA is the most important investigation allowing early diagnosis and precise follow-up [118]. The main features seen in the acute phase of the disease are the presence of disseminated, even, regularly distributed hypofluorescent dark dots (HDDs) in the intermediate and late angiographic phases (Figure 49), accompanied by the usual angiographic signs of stromal choroiditis (Table 1). However, in VKH, ICGA disc hyperfluorescence is much more frequent because of the hyperacute character of the disease. In case of exudative detachments, ICGA shows the same hyperfluorescent pinpoints seen on FA. These signs all regress with treatment. In that regard, ICGA was found to be essential in the follow-up of the disease.

**Figure 50:**
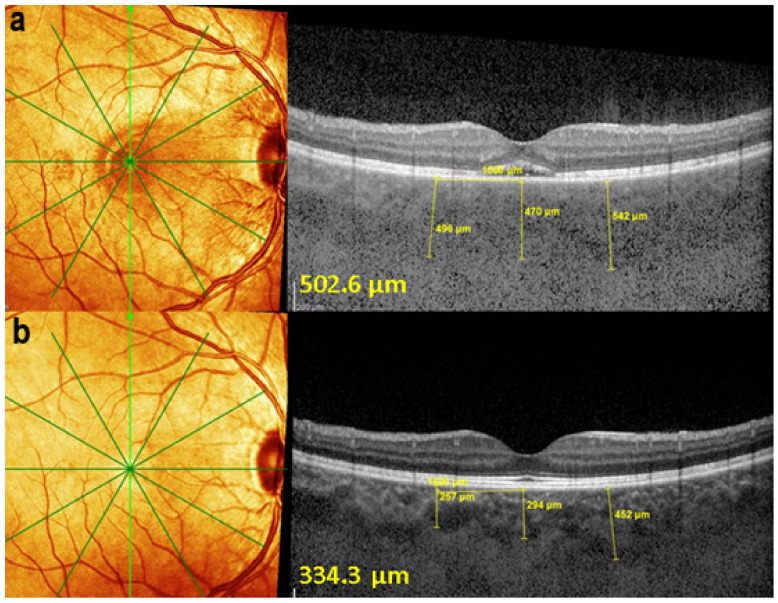
EDI-OCT measuring choroidal thickness in VKH disease. At presentation (**a**), mean subfoveolar thickness is increased to 502.6 µm. After introduction of steroidal and non-steroidal immunosuppression (**b**), thickness was reduced to 334.3 µm.

On FA, serous exudative detachments occurring in the acute early stage appear as multifocal hyperfluorescent pinpoints, showing the leaking points at the level of pigment epithelium also seen on ICGA, with late pooling of the dye in the subretinal space (Figure 48). Optic disc staining also appears in the acute phase. However, no retinal vasculitis is seen. In the chronic phase, FA clearly shows the diffuse retinal pigment epithelium alterations classically appearing as a mixture of window and masking effects as a consequence of the exudative retinal detachment, the limits of which are well shown and are called highwater marks. In the chronic phase, a hyperfluorescent “hot” disc may be the only FA sign indicating inflammatory activity, whereas ICGA can show subclinical choroidal granulomas, a sign of subclinical inflammatory activity. B-can echography is only of interest in case the media are opacified. OCT gives precious information on the evolution of secondary retinal lesions and EDI-OCT measuring choroidal thickening is a complimentary modality allowing follow choroidal inflammation (Figure 50). VKH being principally a stromal choroiditis OCTA is interesting but does clinically contribute to the practical management of VKH.

In order to standardise the appraisal of VKH cases, there have been several attempts to fix diagnostic criteria, the last at the turn of the century [119]. These criteria are however inadequate because the distinction between initial-onset and chronic disease have not been taken into account and new choroidal imaging methods have not been used [120,121]. The use of these criteria has therefore been of limited utility and new criteria are presently being published, taking into account both the distinction of initial-onset and chronic diseases as well as the performing diagnostic tools available to image choroidal inflammation (Table 6).

As far as treatment is concerned, there is a therapeutic window of opportunity after onset of disease, which will avoid substantially the evolution towards chronic disease [122]. Together with prompt initiation of therapy, the other pre-requisite for successful management of VKH disease is the use of first-line steroidal and non-steroidal immunosuppression [123]. Such an approach has drastically reduced the evolution to chronic disease and the development of sunset glow fundus [124]. The management should follow and eradicate subclinical choroidal inflammatory activity thanks to EDI-OCT and ICGA, which will however prolong the treatment, compared to what is usually recommended [125,126,127].

###### Sympathetic Ophthalmia (SO)

Sympathetic ophthalmia (SO) is a bilateral granulomatous uveitis following a penetrating eye injury or repeated intraocular operations. The interval between ocular injury/surgery and the clinical manifestations may vary widely, from a few days to several decades, but 90% of cases occur within one year after the injury [128]. The mechanism is comparable to VKH, auto-sensitivity to an antigenic protein from uveal pigment due to an interruption of the self-tolerance process resulting in bilateral uveitis [129]. Clinical manifestations and histopathologic changes are similar to those of the Vogt-Koyanagi-Harada disease [130,131].

The onset is usually insidious with blurring of vision, loss of accommodation, photophobia, and slight pain in both eyes. The exciting eye is usually chronically inflamed, often phthisical. There is a bilateral granulomatous panuveitis with mutton-fat keratic precipitates, posterior synechiae, a thickened iris, vitreous cells, choroidal infiltration and thickening, perivasculitis, papillitis, and serous detachments, if severe inflammation is present. Dalen-Fuchs nodules are classically described in sympathetic ophthalmia and consist of small yellowish spots, corresponding histopathologically to foci of epithelioid cells containing pigment located between Bruch’s membrane and the retinal pigment epithelium [131]. The natural course of the disease used to lead to a poor visual prognosis, with as many as 70% of the eyes becoming blind in some reports [131].

On ICGA, hypofluorescent dark dots are seen in the intermediate and late angiographic phases with the usual associated ICGA signs of stromal choroiditis (Table 1). FA shows multiple areas of leakage at the level of the retinal pigment epithelium and retinal serous detachments in the acute phase as well as disc hyperfluorescence.

Early treatment with systemic corticosteroids with slow tapering over at least 6–9 months can lead to a good visual outcome [131]. However, as for VKH, aggressive dual steroidal and non-steroidal immunosuppression is recommended [131].

##### 3.2.2. Secondary Stromal Choroiditis

###### Sarcoidosis Chorioretinitis (SARC)

Sarcoidosis is a multisystemic granulomatous disease of unknown aetiology [132]. Lung involvement occurs most frequently, and skin and eye lesions are next in frequency. Distribution is worldwide and prevalence of the disease differs according to racial group, being 10 to 20 times more frequent among blacks than whites. Sarcoidosis is characterised by the presence of noncaseating granulomas in involved organs. Sarcoidosis may affect any ocular tissue. Uveitis is the most common and most serious form of ocular involvement in sarcoidosis and posterior segment inflammation occurs in up to one-third of patients with ocular sarcoidosis [133]. The choroid (and retina) are chance localisations and, in contrast to BRC and VKH where inflammation originates from the choroidal structure, the choroid is just an innocent host of the multisystem inflammatory process. It is therefore classified in secondary stromal choroiditis entities.

Symptoms are diverse and depend on the type of involvement. Patients can complain of blurred vision if cystoid macular edema or vitritis are prominent or if significant anterior uveitis is present [134].

**Figure 51:**
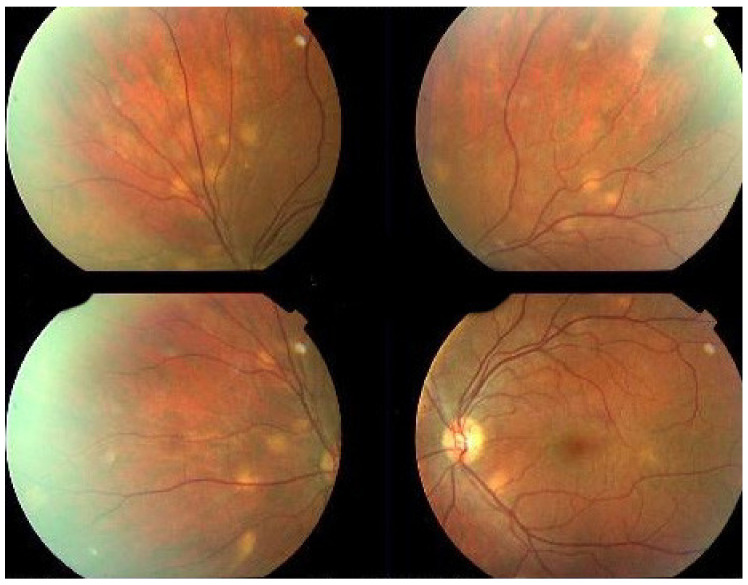
Choroiditis in Sarcoidosis. Unevenly sized randomly distributed fundus lesions.

**Figure 52:**
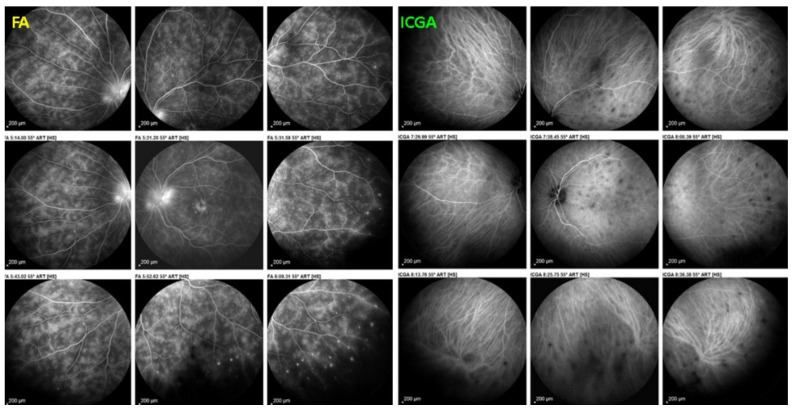
FA and ICGA findings in sarcoidosis chorioretinitis (left eye). FA shows diffuse retinal vasculitis of large and small vessels, papillitis (hyperfluorescent disc), and cystoid macular oedema. ICGA shows HDDs randomly scattered and some areas of fuzzy vessels with most choroidal vessels still well delineated.

**Figure 53:**
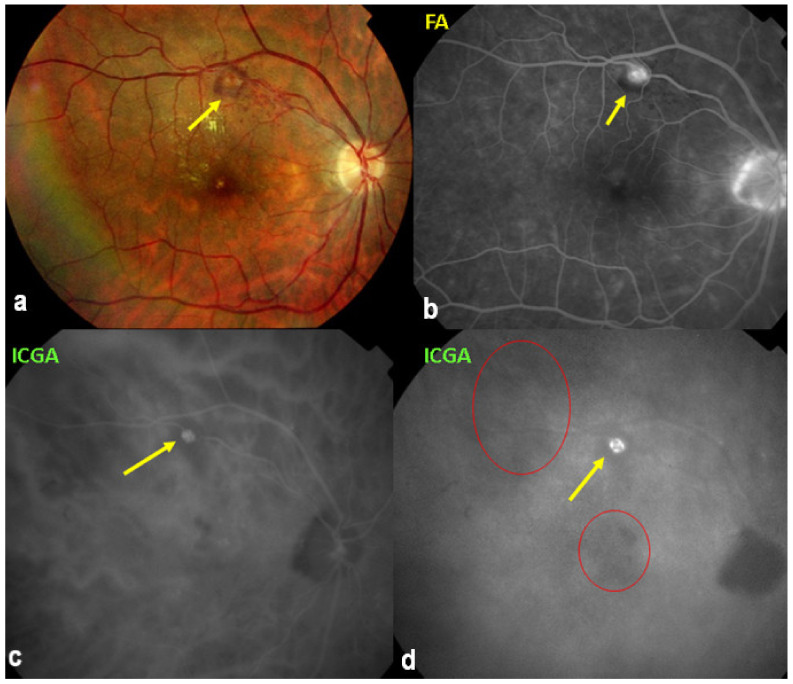
Secondary sarcoid stromal choroiditis. Arterial macroaneurysm (yellow arrows) on fundus photography (**a**), FA (**b**), and ICGA (**c**,**d**). Note fuzziness of choroidal vessels on ICGA (**c**,**d**) and random distribution of hypofluorescent areas (**d**).

Anterior uveitis is often seen in association with choroiditis and retinitis. It is a granulomatous uveitis usually with large mutton-fat KPs, synechiae and iris nodules, and infiltration. Choroiditis and retinal inflammation can occur independently, both being possible random sites of involvement or retinal involvement can be the consequence of underlying foci of choroiditis [135]. Retinal periphlebitis is the most common feature of posterior segment sarcoidosis, vascular sheathing may take the characteristic “candle-wax” appearance. Multifocal choroiditis appears as multiple, pale-yellow, lesions resembling those seen in HLA-A-29 birdshot retinochoroiditis (Figure 51); however, distribution is more at random and this is especially evident on ICGA [134] (Figure 52). A solitary choroidal granuloma is a rarer involvement of the choroid. Vitreous involvement with or without pars plana snowbanking is usual in posterior segment sarcoidosis. Cystoid macular oedema (CMO) and papillitis are common features when chorioretinitis is present. A list of signs suggestive of intraocular sarcoidosis was published by the International Workshop on Ocular Sarcoidosis (IWOS), one of them, retinal microaneurysm, having a high diagnostic value (Figure 53) [136] (Table 7).

For practical monitoring, the most useful imaging modalities are ICGA, FA, and both OCT and EDI-OCT. The signs seen on ICGA are not specific for sarcoidosis but can also be seen in other granulomatous involvements such as ocular tuberculosis. The four-usual stromal ICGA features described on Table 1 are present in different proportions depending on the extent of involvement [137] (Table 1). Choroidal involvement is more random than in primary stromal choroiditis entities (Figure 52). The advantage of ICGA is that it identifies occult choroidal lesions. FA is used to analyse the effects of inflammation at the level of the retina and retinal pigment epithelium. Depending on the type of involvement, it can show disc hyperfluorescence, CMO, or retinal vasculitis (Figure 52) as well as the combination of window effect and masking effect in areas of chorioretinal atrophy after the healing of granulomatous inflammation. OCT allows evaluation and follow-up of the retina, in particular CMO. EDI-OCT measures the thickening of the choroid also depicting large choroidal granulomas. These imaging modalities show an involvement more at random than in primary stromal choroiditis. Use of the dual FA and ICGA scoring system allowed to establish the proportion of retinal versus choroidal involvement in each individual case and makes precise follow-up possible [17,138].

The International Workshop for Ocular Sarcoidosis (IWOS) established diagnostic criteria for ocular sarcoidosis as ocular involvement can occur without other organ involvement [136]. Diagnostic tests routinely practiced help in the diagnosis but have suboptimal sensitivities and specificities. They include chest radiographic and laboratory work-up, including serum angiotensin converting enzyme (ACE) and lysozyme, the latter being much more useful [139]. Anergy in BCG (tuberculin-test)-vaccinated patients and polyclonal antibody activation are other diagnostic helps [139]. An IGRA test (interferon-gamma-release assay) is often performed to exclude tuberculosis as ocular involvement is very similar in both diseases. Gallium scintigraphy shows increased uptake in salivary or lachrymal glands and in the mediastinum and liver, but is now increasingly replaced by PET scan. Bronchoalveolar lavage typically shows a lymphocytosis with an elevated CD4/CD8 ratio.

Ocular sarcoidosis is quite sensitive and responsive to corticosteroids that are often given in combined forms, including ocular drops, sub-Tenon’s injection, and systemic therapy. Indication for therapy depends on the severity of the lesions. In severe forms of disease or in case of corticosteroid intolerance, an immunosuppressive agent such as mycophenolic acid (Myfortic^®^) or other needs to be added, which will also allow to achieve a steroid-sparing effect. Anti-TNF-alpha agents are an alternative, but exclusion of exposure to Mycobacterium tuberculosis by IGRA testing is mandatory [140].

## 4. Discussion—Conclusions

In the last 25 years, the field of posterior uveitis and in particular non-infectious choroiditis has evolved enormously with the development of multimodal imaging, starting with ICGA in the mid-nineties, followed by SD-OCT and EDI-OCT and to a lesser extent OCTA. Most clinical pictures had been described phenotypically previously by sharp observers. While the clinical descriptions were very accurate, the explanations of disease mechanisms remained hypothetical and the structures at the origin of the diseases were often not correctly identified. Hence, it was difficult to go beyond the morphological description and to classify the diseases in an adequate manner according to their clinicopathology and this was especially true of non-infectious choroiditis. Today a more precise classification of inflammatory disease of the choroid has become possible and has been exposed in the present review and by others. By better understanding the mechanisms at the origin of inflammatory diseases of the choroid and by having better tools to follow their evolution and to monitor their treatment, the quality of management has substantially improved.

## Figures and Tables

**Table 1:** ICG angiographic signs in stromal choroiditis.

1. Hypofluorescent dark dots (HDDs)
2. Indistinct choroidal vessel (Fuzziness of choroidal vessels)
3. Diffuse late choroidal hyperfluorescence (partially hiding HDDs)
4. ICGA disc hyperfluorescence (in severe choroiditis)

**Table 2:** Multimodal imaging of MEWDS lesions and clinicopathological explanation.

BAF	Hyperfluorescence (increased exposure of RPE lipofuscin following loss of outer photoreceptor segments)
ICGA	Hypofluorescence (intermediate and late phase) (choriocapillaris non-perfusion of endcapillary portions)
SD-OCT	Loss of photoreceptor outer segments
OCTA	No sign of choriocapillaris drop out (OCTA is incapable to image low-flow endcapillary vessels)
FA	Faint hyperfluorescence (possibly due to outer retina ischaemia)

BAF = Blue-light fundus autofluorescence; ICGA = indocyanine green angiography; SD-OCT = spectral domain optical coherence tomography; OCTA = OCT angiography; FA = fluorescein angiography.

**Table 4:** Multimodal imaging of idiopathic MFC lesions and clinicopathological explanation.

BAF	Hyperautofluorescence (exposure of RPE lipofuscin due to loss of outer segments) (occult lesions in active disease)
	Hypoautofluorescence of atrophic lesions due to previous episodes
ICGA	Hypofluorescent areas (atrophic areas of previous episodes + active choriocapillaritis (non-perfusion)) of new occult active areas
OCT	Loss of photoreceptor outer segments in occult active areas ± chorioretinal atrophic areas; also hyperreflective clumps
OCTA	Choriocapillaris drop-out (less sensitive than ICGA); also useful to identify CNV
FA	Very early hypofluorescence followed by late hyperfluorescence (full atrophic areas of previous episodes/ window effect)
	Active lesions occult, barely visible, faint late hyperfluorescence unless extensive choriocapillaritis

BAF = blue light fundus autofluorescence; ICGA = indocyanine green angiography; SD-OCT = spectral domain optical coherence tomography; OCTA = OCT angiography; FA = fluorescein angiography.

**Table 5:** Global diagnostic criteria for HLA-A29 birdshot retinochoroiditis [101].

1. Presence of vitritis in one or both eyes (required)
2. Presence of retinal vasculitis in one or both eyes (required)
3. Stromal choroiditis, as evidenced by ICGA, in both eyes (required)
4. HLA-A29 antigen positivity (required)
5. Visual field anomalies in one or both eyes (supportive)
6. Absence of extra-ocular inflammatory site (supportive)
7. Presence of rice-shaped depigmented “birdshot lesions” (BRC fundus lesions) (strongly supportive but not required)

**Table 6:** Diagnostic criteria for initial onset VKH disease [121].

1. No ocular trauma or surgery preceding onset of disease *
2. Bilateral involvement (verified with ICGA and/or EDI-OCT) *
3. Exclusion of other infectious, inflammatory, or masquerading entities, in particular other stromal choroiditis entities (i.e., tuberculosis, sarcoidosis, or syphilis) *
4. Diffuse choroiditis evidenced by ICGA and/or EDI-OCT *
5. Signs and symptoms of less than four-weeks’ duration *
6. Absence of clinical findings compatible with chronic disease (i.e., sunset glow fundus or integumentary signs (vitiligo, alopecia, and poliosis) *
7. Exudative retinal detachments (evidenced by pooling and pinpoint leaking points on FA and ICGA) (very helpful criterion when present)
8. Disc hyperfluorescence (helpful criterion)
9. Neurological/auditory findings (meningismus, tinnitus, acute hearing loss) (helpful criterion)

* Essential criteria.

**Table 7:** List of IWOS clinical signs suggestive of ocular sarcoidosis [136].

1. Mutton-fat KPs (large and small) and/or iris nodules at pupillary margin (Koeppe) or in stroma (Busacca)
2. Trabecular meshwork (TM) nodules and/or tent-shaped peripheral anterior synechiae (PAS)
3. Snowballs/string of pearls vitreous opacities.
4. Multiple chorioretinal peripheral lesions (active and atrophic)
5. Nodular and/or segmental peri-phlebitis (± candle-wax drippings) and/or macroaneurysm in an inflamed eye
6. Optic disc nodule(s)/granuloma(s) and/or solitary choroidal nodule
7. Bilaterality (assessed by clinical examination or investigational tests showing subclinical inflammation).

## Data Availability

Please refer to corresponding author.
